# C-MYC Transcriptionally Amplifies SOX2 Target Genes to Regulate Self-Renewal in Multipotent Otic Progenitor Cells

**DOI:** 10.1016/j.stemcr.2014.11.001

**Published:** 2014-12-11

**Authors:** Kelvin Y. Kwan, Jun Shen, David P. Corey

**Affiliations:** 1 Department of Cell Biology & Neuroscience, Rutgers University, Piscataway, NJ 08854, USA; 2 Howard Hughes Medical Institute, Department of Neurobiology, Harvard Medical School Boston, MA 02115, USA; 3 Department of Pathology, Brigham and Women’s Hospital, Boston, MA 02115, USA

## Abstract

Sensorineural hearing loss is caused by the loss of sensory hair cells and neurons of the inner ear. Once lost, these cell types are not replaced. Two genes expressed in the developing inner ear are *c-Myc* and *Sox2*. We created immortalized multipotent otic progenitor (iMOP) cells, a fate-restricted cell type, by transient expression of C-MYC in SOX2-expressing otic progenitor cells. This activated the endogenous C-MYC and amplified existing SOX2-dependent transcripts to promote self-renewal. RNA-seq and ChIP-seq analyses revealed that C-MYC and SOX2 occupy over 85% of the same promoters. C-MYC and SOX2 target genes include cyclin-dependent kinases that regulate cell-cycle progression. iMOP cells continually divide but retain the ability to differentiate into functional hair cells and neurons. We propose that SOX2 and C-MYC regulate cell-cycle progression of these cells and that downregulation of C-MYC expression after growth factor withdrawal serves as a molecular switch for differentiation.

## Highlights

• A single factor, C-MYC, induces self-renewal in SOX2-expressing otic progenitors
• C-MYC transcriptionally amplifies SOX2 target genes
• SOX2 modulates transcription of cell-cycle genes
• Immortalized multipotent otic progenitors can differentiate into otic cell types

## Graphical Abstract

**Figure:**
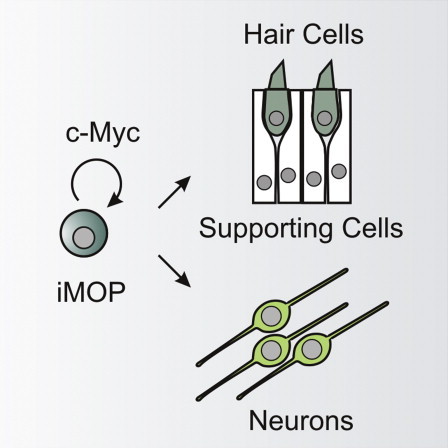

## Teaser

In this work, Kwan and colleagues generated an immortalized multipotent otic progenitor (iMOP) cell by transient expression of C-MYC in SOX2-expressing otic progenitor cells. The procedure activated endogenous C-MYC expression in the cells and amplified existing SOX2-dependent transcripts to promote self-renewal. Downregulation of C-MYC expression after growth factor withdrawal resulted in a molecular switch from self-renewal to otic differentiation.

## Introduction

The six sensory organs of the inner ear—the cochlea, utricle, saccule, and three semicircular canals—mediate our ability to hear and balance. Within these organs, sensory hair cells mediate the conversion from mechanical to neural signals, releasing neurotransmitter onto neurons of the eighth nerve. Built for exquisite sensitivity, hair cells have a high metabolic demand and delicate mechanosensory hair bundles. A variety of insults, such as loud noises and ototoxic drugs, can cause hair cell death. They can also cause acute loss of afferent nerve terminals and delayed degeneration of the auditory nerve (Kujawa and Liberman, 2009). Degeneration of hair cells and neurons significantly contributes to hearing loss, as these cells are not replaced. To regenerate auditory hair cells and neurons, we must understand how progenitor cells give rise to these cell types.

*Sox2* and the *Myc* family of transcription factors are crucial for the proper development of inner ear hair cells and neurons. Human mutations in *Sox2* cause anophthalmia, a severe eye malformation, and bilateral sensorineural hearing loss (Fantes et al., 2003; Hagstrom et al., 2005). Mouse mutants that express low levels of SOX2 in the inner ear have fewer cochlear hair cells and neurons (Kiernan et al., 2005; Puligilla et al., 2010). *Myc* genes, including *c-Myc* and *N-Myc*, are expressed in the inner ear (Domínguez-Frutos et al., 2011; Kopecky et al., 2011). Deletion of *N-Myc* in the developing inner ear causes reduced proliferative growth, and abnormal morphology and differentiation of both sensory and nonsensory cells (Domínguez-Frutos et al., 2011; Kopecky et al., 2011).

Studies aimed at producing new hair cells and otic neurons have used embryonic stem cells (ESCs) or induced pluripotent stem cells (iPSCs). iPSCs are generated by converting somatic cells into pluripotent stem cells that possess properties of both self-renewal and pluripotency (Takahashi and Yamanaka, 2006). This involves transient expression of *c-Myc*, *Sox2*, *Klf4*, and *Oct4* to activate expression of the endogenous factors. The endogenous factors function to promote self-renewal, maintain pluripotency, and prevent differentiation. Among the four transcription factors used to generate iPSCs, C-MYC and SOX2 have been implicated in maintaining self-renewal in ESCs (Cartwright et al., 2005). *Sox2* is also essential for maintaining multipotency in neural stem cells (Suh et al., 2007), and knockout or knockdown of *Sox2* in ESCs results in differentiation (Ivanova et al., 2006). Although *c-Myc* is dispensable for direct reprogramming of somatic cells into pluripotent cells, inclusion of *c-Myc* increases the number of reprogrammed cells and accelerates the formation of iPSCs (Wernig et al., 2008). Recent genome-wide binding studies implicated C-MYC as a global transcription amplifier (Lin et al., 2012; Nie et al., 2012), providing an elegant explanation of the diverse roles of C-MYC in reprogramming and in various cellular functions.

We exploited C-MYC to activate the endogenous *c-Myc* gene and enhance gene expression in neurosensory cell types. By doing so, we derived a self-renewing immortalized multipotent otic progenitor (iMOP) line from SOX2-expressing neurosensory precursors of the inner ear. We show that the endogenous C-MYC binds to most of the same promoters as SOX2 and amplifies transcripts that promote cell-cycle progression. This enhanced expression contributes to self-renewal but allows iMOP cells to retain their capacity to differentiate into hair cells, supporting cells and neurons.

## Results

### Induction of Self-Renewal by Transient C-MYC Expression

During embryonic development of the murine cochlea, progenitors begin exiting the cell cycle at embryonic day 12.5 (E12.5). Terminal mitosis spreads in a wave-like manner from the apex to the base of the cochlea, completing cell-cycle exit by E14.5 (Lee et al., 2006; Ruben 1967). Progenitors stop dividing and express the cell-cycle inhibitor *Cdkn1b* (*p27*^*KIP*^) at E14.5 to initiate differentiation (Chen and Segil, 1999). We obtained and dissociated cochleas from E11.5–12.5 embryos into single cells. Dissociated cells were cultured in defined medium supplemented with basic fibroblast growth factor (bFGF). Cells were plated on untreated tissue culture dishes to produce both adherent cells and colony-forming cells (Figure S1A available online). Colony-forming otic cells (known as otospheres) were enriched by gently superfusing the cultures and collecting the suspension of otospheres (Figure S1B). In 50 otospheres examined, ∼60% of cells expressed detectable levels of SOX2, and in culture they incorporated the nucleotide analog 5-ethynyl-2′-deoxyuridine (EdU), indicating they were dividing (Figure S1C).

Cells from postnatal cochleas can form three types of otospheres: solid, transitional, and hollow (Diensthuber et al., 2009). Otospheres from postnatal vestibular and auditory organs contain dividing cells that can become neurons and sensory cells (Oshima et al., 2007). To identify spheres derived from embryonic cochlea, we dissociated cochleas and cultured cells for 3–5 days until otospheres were observed. Primary otospheres were fixed, embedded in plastic, serially sectioned, and observed by transmission electron microscopy (TEM). In 100 otospheres examined, we found cells in all sections, suggesting that embryonic primary otospheres are similar to solid spheres from postnatal inner ear organs (Figure S1D).

**Figure 1:**
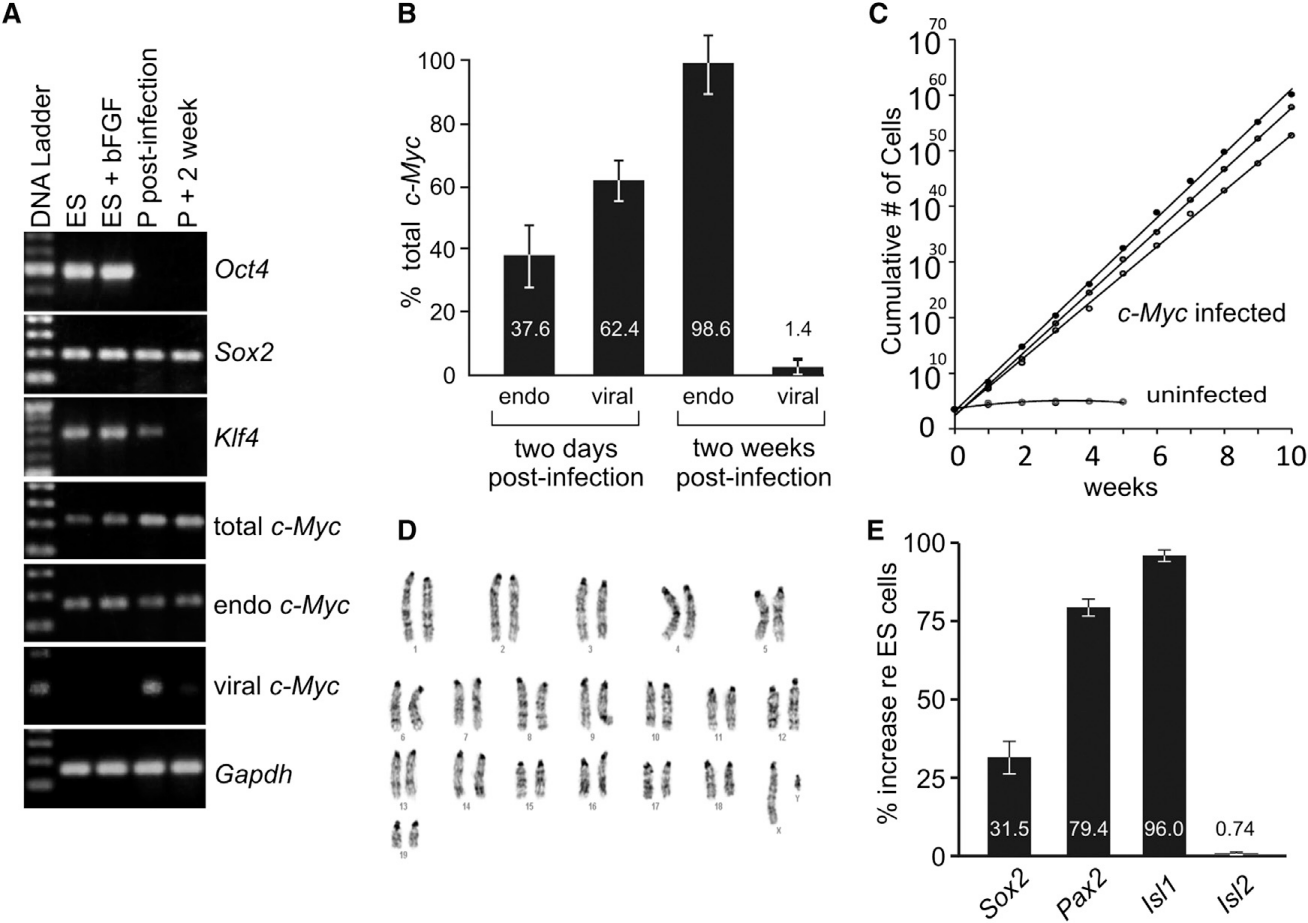
Determining Properties of Self-Renewal and Pluripotency in iMOP Cells (A) RT-PCR of stem cell factors in ESCs and progenitor cells (P) revealed the presence of *Sox2* in all conditions, and transient expression of *Klf4* and *c-Myc* after infection with a *c-Myc* retrovirus. The pluripotency gene *Oct4* was present in ESCs cultured either in ESC media or in progenitor-cell media, but was absent in progenitor cells. (B) Real-time PCR to detect the presence of both endogenous and viral *c-Myc* transcripts 2 days or 2 weeks after infection with the *c-Myc* retrovirus. After 2 weeks of culturing, less than 2% of the total *c-Myc* transcript was the viral form and the great majority was endogenous *c-Myc*, apparently due to silencing of the retroviral *c-Myc* gene. Independently derived cultures were used as replicates (n = 3); error bars are depicted as SEM. (C) Growth curves. iMOP cells continuously divided, whereas primary cells had a limited capacity to proliferate. The doubling time of the iMOP cells was ∼18 hr. (D) Karyotype of a clonal iMOP cell line. iMOP cells maintained a normal ploidy with 19 autosomes and a pair of XY chromosomes. (E) Real-time PCR of iMOP cells showed continued expression of proneurosensory transcripts *Sox2*, *Pax2*, and *Isl1*. *Isl2* served as a negative control. Independently derived cultures were used as replicates (n = 3); error bars are SEM. See also Figures S1–S4 and Table S3.

We next sought a way to promote long-term self-renewal, and asked whether a single gene, *c-Myc*, could amplify the underlying gene-expression profile to promote self-renewal. We used a retrovirus to introduce exogenous C-MYC into SOX2-expressing neurosensory precursors and assessed activation of endogenous C-MYC in these cells. Primers were designed to detect and distinguish total *c-Myc*, endogenous *c-Myc*, and transgenic *c-Myc* transcripts. As controls, we used ESCs cultured under normal conditions and in otic progenitor media used for culturing SOX2-expressing otospheres to detect all four transcription factors that induce pluripotency. We found that expression of the four transcription factors was not altered in ESCs. In progenitor cells, *Oct4*, a crucial factor for pluripotent ESCs and iPSCs (Takahashi and Yamanaka, 2006), was not detected, whereas *Sox2* was detected in all samples. This suggests that the iMOP cells, unlike iPSCs, are not pluripotent but are fate restricted. Viral *c-Myc* was transiently upregulated along with *Klf4* 2 days after infection but decreased after the cells were cultured for 2 weeks. Endogenous *c-Myc* and total *c-Myc* were present in iMOP cells and did not show a large upregulation in transcript levels even after integration of the *c-Myc* retrovirus (Figure 1A). To determine the contribution of endogenous and viral *c-Myc* to total *c-Myc* levels, we performed quantitative RT-PCR (qPCR) and normalized the transcript levels to total *c-Myc* levels. At 2 days postinfection, endogenous and viral *c-Myc* represented 37.6% and 62.4% of total *c-Myc*, respectively. At 2 weeks postinfection, viral *c-Myc* was 1.4% of total, indicating that the *c-Myc* retrovirus had been silenced (Figure 1B), similar to what was previously observed in factor-based reprogramming of iPSCs (Hotta and Ellis, 2008). To compare *c-Myc* transcript levels with endogenous levels in the inner ear, we performed qPCR and normalized the transcript levels to E12.5 cochleas. ESCs and *c-Myc*-infected-progenitor cells showed increased *c-Myc* transcript compared with uninfected progenitor cells (Figure S1E).

To select for dividing cells that still maintained their otic identity, we cultured cells in defined medium with bFGF. bFGF promotes the proliferation of inner ear epithelia cultures (Zheng et al., 1997) and also induces otic cell identity (Groves and Bronner-Fraser, 2000). Mesenchymal and pluripotent stem cells that were previously used to generate hair cells were also treated with bFGF to propagate cultures and induce otic cell fate (Hu and Corwin, 2007; Oshima et al., 2010; Koehler et al., 2013). We continuously expanded the cells in bFGF medium for up to 36 months to maintain otospheres. Otosphere clones derived from single cells were compared with ESCs (Figures S2A and S2B). To ensure that these were proliferative proneurosensory cells that retained a restricted identity, we assessed the expression of endogenous alkaline phosphatase, a marker for pluripotent cells (Stadtfeld and Hochedlinger, 2010). Unlike pluripotent ESCs, progenitor cells derived from otospheres did not express endogenous alkaline phosphatase and were unlikely to be pluripotent (Figures S2C and S2D). To determine the expression level of proneurosensory markers relative to the inner ear, we performed qPCR for *Sox2*, *Pax2*, and *Isl1* using progenitors and *c-Myc*-infected progenitor cells. Transcript levels were normalized to E12.5 embryonic cochlea. Both cell types retained expression of all three proneurosensory markers (Figure S2E). We thus named the *c-Myc*-infected progenitor cells *i*mmortalized *m*ultipotent *o*tic *p*rogenitor (iMOP) cells.

To determine the proliferative capacity of iMOP cells after transient C-MYC expression, we clonally derived and expanded three primary otospheres and three iMOP cell lines. Cells (10^4^) were passaged and the cumulative cell counts were tabulated weekly. Primary cells from otospheres initially expanded exponentially (Figure S3), but stopped dividing after 5 weeks in culture, whereas iMOP cells continued to divide at a much faster rate during 10 weeks in culture (Figure 1C), doubling in ∼18 hr at a rate similar to that observed for ESCs.

Many immortalized cell types, such as ESCs and iPSCs, acquire chromosomal abnormalities in continuous culture. The karyotype of ESCs is well known to be metastable, with 50%–60% of cells showing a normal karyotype and a high degree of aneuploidy (Rebuzzini et al., 2008). For cells in culture, aneuploidy presumably would be disadvantageous and the cells might not continue growing. We examined one of the clonal iMOP cell lines for chromosomal stability. It displayed a normal karyotype with 19 pairs of autosomes and an XY chromosome (Figure 1D) and had a distribution of chromosome numbers similar to that found for ESCs (n = 40) (Figure S4). Thus, iMOP cells showed a genomic stability similar to that of pluripotent ESCs. To determine whether the cells still retained transcripts for otic neurosensory markers, we conducted qPCR. The proneurosensory transcripts *Sox2*, *Pax2*, and *Isl1* were enriched relative to pluripotent ESCs (by 31.5%, 79.4%, and 96.0%, respectively), whereas the negative control *Isl2*, a marker for differentiating sensory and nonsensory cells of the inner ear (Huang et al., 2008), showed no significant changes in transcript levels (<1%; Figure 1E). Infection of otic progenitors with a *c-Myc* retrovirus apparently activates the endogenous *c-Myc* before silencing itself, and allows for prolonged proliferation of cells that retain otic neurosensory transcripts.

### Transcriptional Amplification of SOX2 Target Genes by C-MYC

Because the endogenous *c-Myc* transcript accounts for most (98.6%) of the total *c-Myc* transcript (Figure 1B), we asked whether endogenous C-MYC and SOX2 are responsible for self-renewal in iMOP cells. At early stages of otic development, SOX2 could maintain or establish neurosensory cell fate and promote proliferation, while C-MYC transcriptionally amplified SOX2 target genes. To identify their genome-wide targets in iMOP cells, we used chromatin immunoprecipitation sequencing (ChIP-seq) with antibodies against C-MYC or SOX2. In parallel, we defined promoter regions around known transcriptional start sites (TSSs) using reads obtained from RNA polymerase II (POLII) ChIP-seq in iMOP cells. Binding sites of SOX2 and endogenous C-MYC at promoter regions were determined by enrichment of sequences within the ±5 kb region of the TSS. Genes with both RNA POLII and the transcription factors bound in the promoter region were considered target genes.

**Figure 2:**
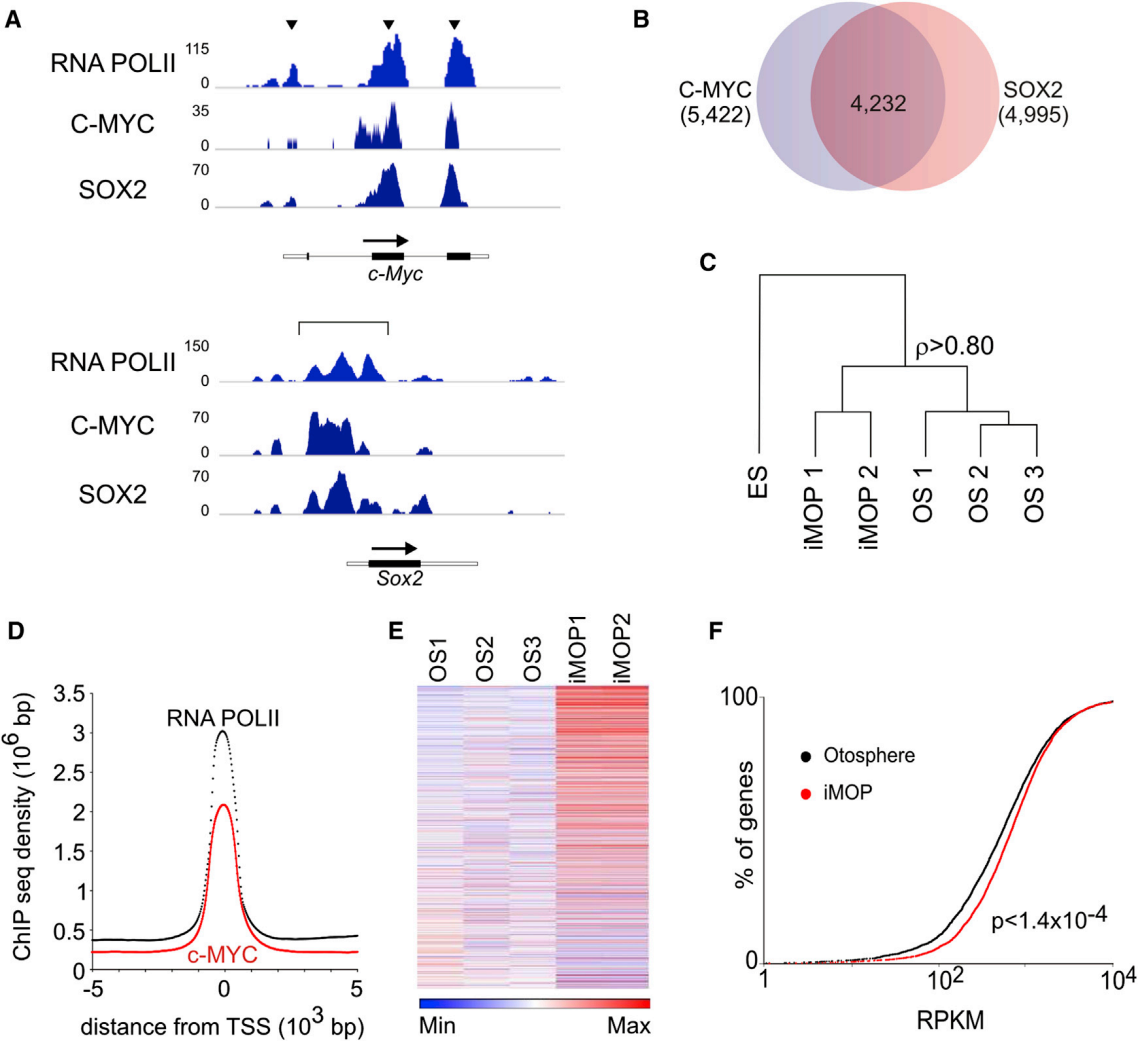
Binding Sites of C-MYC and SOX2 and Target Gene Transcript Levels in iMOP cells (A) Differential binding of C-MYC and SOX2 near RNA-POLII-binding regions at the *c-Myc* and *Sox2* genes shows enrichment patterns near promoters of *c-Myc* splice variants and *Sox2*. (B) C-MYC binds to ∼85% (4,231/4,994) of SOX2 target genes. (C) Hierarchical clustering analysis of RNA-seq samples from ESCs, iMOP cells, and otospheres (OS) using all expressed genes. Numbered samples denote RNA-seq data from individual cell lines. iMOP cells and otospheres show a high degree of correlation (Spearman’s rank correlation ρ > 0.80) (D) Quantile normalized RNA POLII and C-MYC ChIP-seq densities map ±5 kb around the TSS. (E) Relative gene expression from SOX2 and C-MYC target genes. RPKM are plotted for each gene from three otosphere samples (OS) and two iMOP samples. RPKM are plotted for each gene and the relative read counts are denoted by color. The maximum fold increase is 5,540 and the mean and median fold increases are 4.7 and 1.3, respectively. (F) SOX2 and C-MYC target genes. The cumulative distribution of normalized reads from averaged otosphere and iMOP samples is shown. C-MYC and SOX2 target genes with 1–10^4^ RPKM from otosphere and iMOP samples were plotted. The difference between the two samples is statistically significant using Welch’s two-tailed t test (p < 1.4 × 10^−4^). See also Tables S1 and S2.

Mapping the overlapping RNA POLII-, C-MYC-, and SOX2-binding sites on the *c-Myc* and *Sox2* genes, we observed three RNA POLII peaks for the *c-Myc* gene (Figure 2A, arrowheads). These correspond to the promoter regions of three known *c-Myc* splice variants. In the promoters of the *c-Myc* gene, both C-MYC and SOX2 were bound. For the *Sox2* gene, RNA POLII occupied a broad peak in the promoter. C-MYC and SOX2 both occupied the same promoter region on the *Sox2* gene (Figure 2A). These results suggest that C-MYC and SOX2 occupy each other’s promoter regions and may auto- and cross-regulate RNA-POLII-dependent transcription at each other’s promoter.

To identify all the genes regulated by C-MYC and SOX2 in iMOP cells, we defined SOX2 and C-MYC target genes based on binding of RNA POLII and the two transcription factors in the promoter region. A total of 4,994 target genes were identified as direct targets of SOX2 while 5,422 genes were direct targets of C-MYC (Table S1). By comparing the overlap of promoter binding regions, we found that 4,231 genes or ∼85% of SOX2 target gene promoters were also occupied by C-MYC (Figure 2B).

We predicted that by amplifying the existing SOX2 transcriptional program, C-MYC helps retain cellular processes attributed to SOX2 in iMOP cells. To determine how much iMOPs and primary otospheres differ, we performed a hierarchical clustering analysis on all detectable transcripts from RNA sequencing (RNA-seq) samples obtained from ESCs, on two independently derived iMOP cell lines, and on three independently derived otospheres (Figure 2C). Based on gene expression, otospheres and iMOP cells cluster together rather than with ESCs. RNA-seq samples from otospheres and iMOP cells showed a high Spearman’s rank correlation coefficient of ρ > 0.8 (where 1.0 suggests perfect correlation). At the level of the transcriptome, iMOP cells appear similar to cells from primary otospheres and only a subset of transcripts are differentially expressed.

To determine whether C-MYC occupies the promoter and enhancer regions near the TSS, we assessed binding sites of RNA POLII and C-MYC by mapping the quantile normalized RNA POLII ChIP-seq density ±5 kb around the TSS. The majority of C-MYC binding was within 1 kb of the TSS and had a similar distribution to RNA POLII (Figure 2D). This suggests that C-MYC binds in the promoter proximal sites near the TSS, genome wide. To see whether C-MYC transcriptionally amplifies the SOX2 target genes in iMOP cells relative to primary otospheres, we selected normalized reads from the 4,231 genes that were both C-MYC and SOX2 targets. We ranked and compared individual target genes from two iMOP cell lines and three primary otospheres. To display the relative changes for each gene, we normalized reads from C-MYC and SOX2 target genes by subtracting the median read from each gene and dividing by the median absolute deviation. These reads were compiled on a heatmap, which revealed that transcripts from C-MYC and SOX2 target genes displayed a graded degree of amplification in iMOP cells (shown in red) relative to primary otospheres (Figure 2E).

To understand the distribution and extent of the transcript increase in iMOPs compared with otospheres, we plotted the cumulative distribution of reads per kilobase per million (RPKM) from individual genes. A nonlinear, sigmoidal distribution of transcripts was observed in primary otospheres. A similar distribution was also observed in iMOP cells, except that the global distribution of transcripts in iMOP cells was shifted to the right (p < 1.5 × 10^−9^ in Welch’s two-tailed t test; Figure 2F). The global increase in transcripts of C-MYC and SOX2 targets in iMOP cells relative to primary otospheres is consistent with the universal and nonlinear amplification by C-MYC of this subset of actively transcribed genes.

### iMOP Cells Adopt a Molecular Signature that Promotes Proliferation

**Figure 3:**
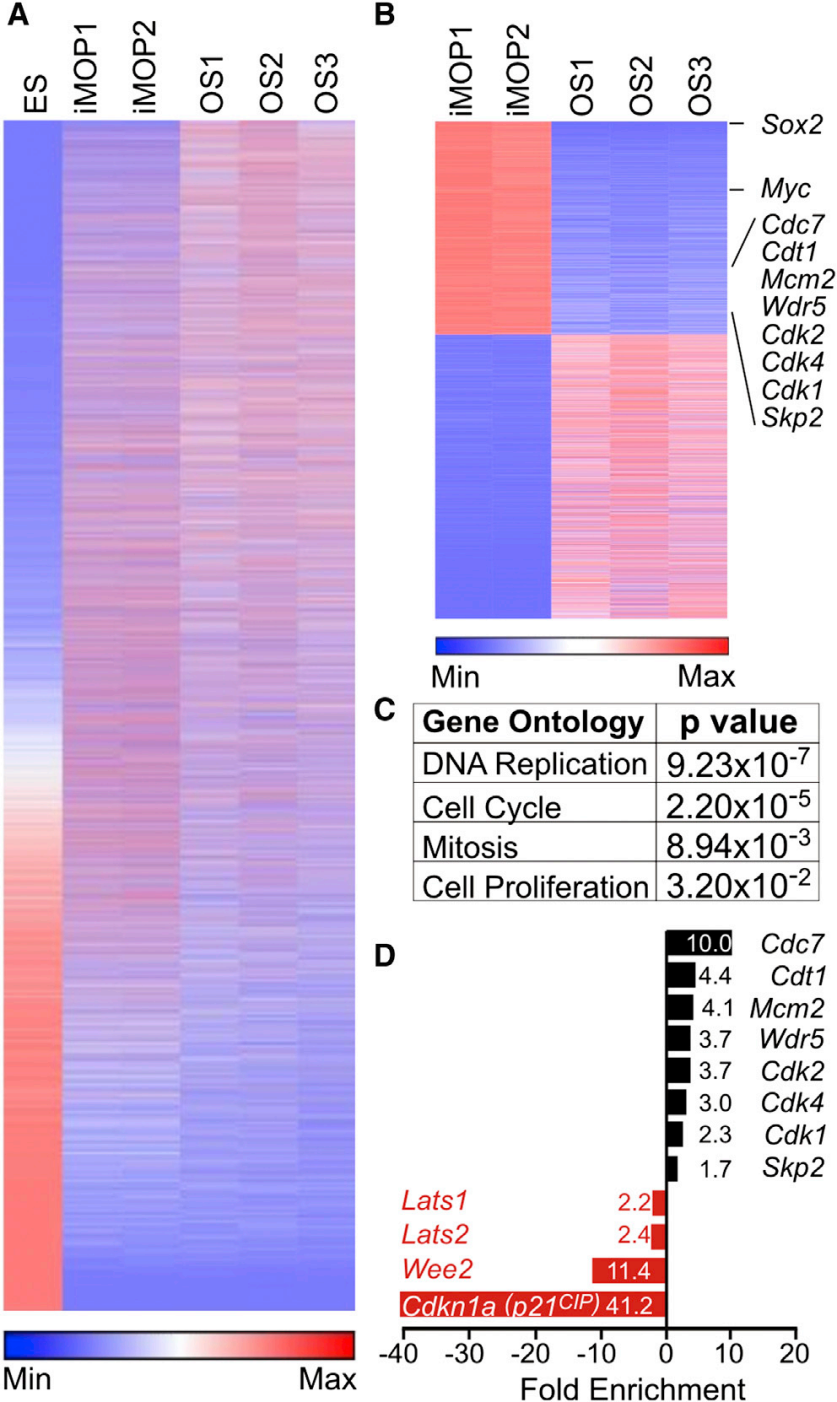
Transcriptome Comparison of iMOP and Otosphere Cells (A) Heatmap of detectable transcripts from ESC, iMOP, and otosphere (OS) RNA-seq samples, plotted with relative read counts depicted by color. (B) Differentially expressed genes from pairwise comparison between averaged iMOP samples and otospheres. Genes that displayed statistically significant differences (p < 0.05) were identified. Individual C-MYC and SOX2 target genes are noted on the heatmap on the right. (C) Functions of differentially expressed genes (p < 0.05) from iMOP and otosphere samples. The table shows pertinent biological processes based on Gene Ontology analysis and the associated p value. (D) Differential expression of significantly altered genes (p < 0.05) from Gene Ontology analysis from pairwise comparison of iMOP and OS samples.

To determine the consequences of amplification of this subset of genes, we wished to identify all the genetic factors attributed to self-renewal, including genes both directly and indirectly affected by C-MYC. We performed RNA-seq on proliferating iMOP cells and primary otospheres cultured with bFGF. To identify these transcripts, we compared ESC, iMOP, and otosphere samples. Gene expression in iMOP cells was much more similar to that in cells derived from otospheres than to ESCs (Figure 3A). We performed a pairwise comparison between iMOP cells and otosphere samples and identified transcripts that were significantly different between iMOP and otosphere samples (p < 0.05). Reads from individual genes were plotted on a heatmap to show the relative changes between iMOP cells and otospheres (Figure 3B). The upper and lower portions of the heatmap showed highly upregulated and downregulated genes. We observed an ∼34-fold increase in *c-Myc* (p < 10^−154^) and an ∼5,700-fold increase in *Sox2* (p < 10^−180^) in iMOP cells relative to otospheres. Selected upregulated genes in iMOP cells (labeled) were direct targets of both C-MYC and SOX2 as determined by ChIP-seq. To determine all of the genes that promote self-renewal in iMOP cells, we identified all of the differentially expressed genes and categorized them based on Gene Ontology. Significant functional groups included genes for DNA replication, cell cycle, mitosis, and cell proliferation (Figure 3C). One of these genes was *Wdr5*, a WD-repeat-containing protein that is essential for histone H3K4 methylation and mediates self-renewal in ESCs (Ang et al., 2011). *Wdr5* showed an ∼5.9-fold increase (p < 10^−5^) in iMOP cells compared with otospheres. Many cyclin-dependent kinases were also identified. *Cdk1* (2.3-fold increase; p < 10^−21^) is part of a highly conserved cyclin-dependent protein-kinase complex that is essential for G1/S and G2/M phase transitions of the eukaryotic cell cycle. *Cdk2* (3.7-fold increase; p < 10^−34^) is another cyclin-dependent kinase that allows the G1/S transition. *Cdk4* (2.1-fold; p < 10^−43^) promotes progression through the G1 phase of the cell cycle. Other genes, such as *Cdc7* (10-fold; p < 10^−65^), *Mcm2* (4.1-fold; p < 10^−58^), *Cdt1* (4.4-fold; p < 10^−50^), and *Skp2* (1.7-fold; p < 10^−8^), regulate the initiation of DNA replication.

Many of the downregulated genes function to inhibit cell-cycle progression. We observed a decrease in the cyclin-dependent kinase inhibitor *Cdkn1a* (p21^CIP^) (−41-fold; p = 0). Similarly, both *Lats1* (−2.2-fold; p < 10^−31^) and *Lats2* (−2.4-fold; p < 10^−29^), tumor suppressors that negatively regulate cell-cycle progression, showed a decrease in transcript levels. *Wee2* (−11.4-fold; p < 3 × 10^−4^), a gene that encodes a kinase that phosphorylates and inhibits CDK1, was also decreased in iMOP cells. The altered levels of both positive and negative regulators of cell-cycle progression could contribute to the increased proliferative capacity of iMOP cells relative to primary otospheres. This constellation of signature cell-cycle genes may contribute to the ability of iMOP cells to continually divide and retain their otic cell identity.

**Figure 4:**
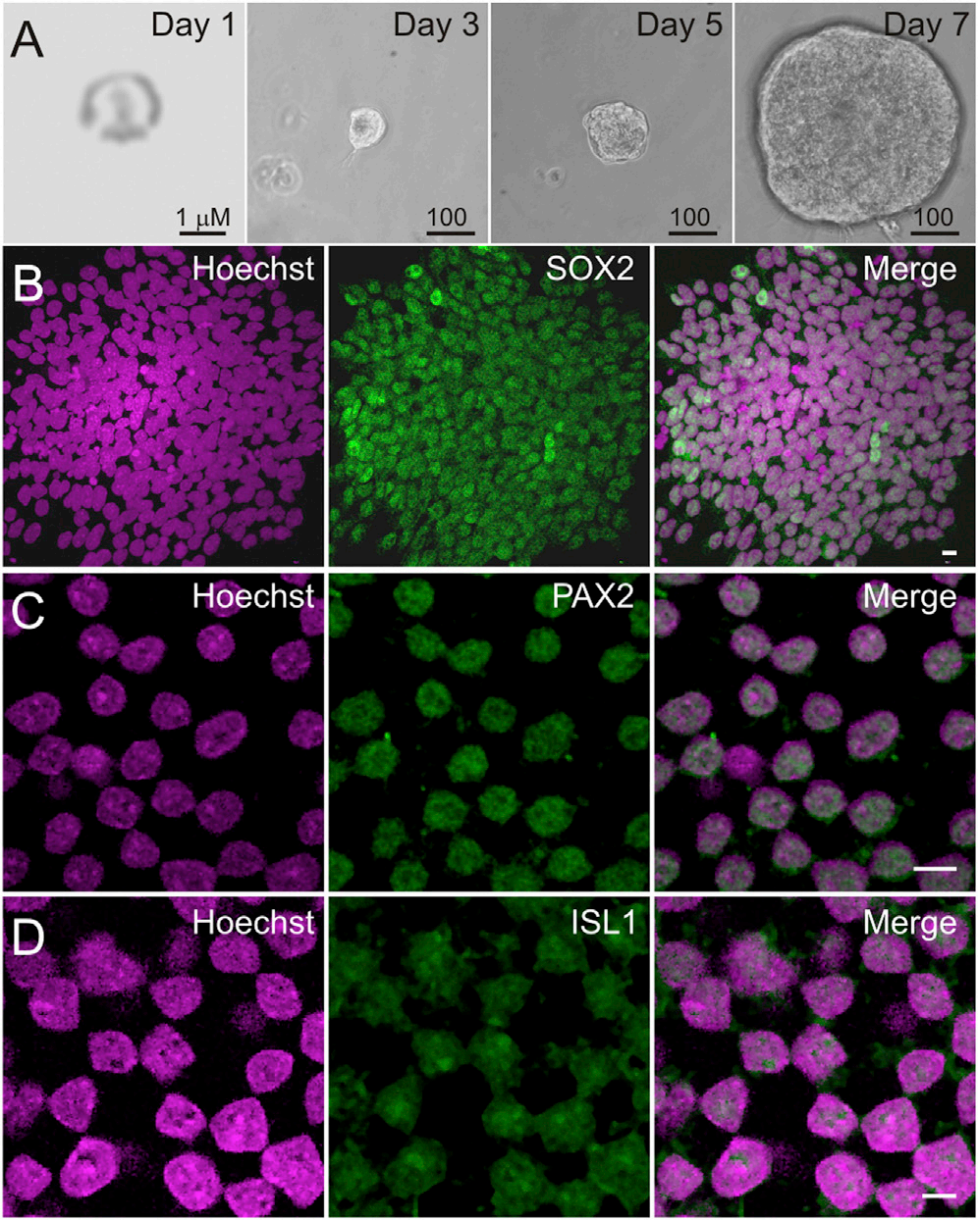
Immobilization of a Single iMOP Cell to Test for Symmetrical Self-Renewal Hoechst stain labels nuclei and antibody labeling reveals SOX2, PAX2, and ISL1 expression. (A) Tracking of a single cell embedded in Matrigel showed colony formation progressing to a multicellular otosphere. (B) iMOP colony derived from a single cell. Most cells expressed SOX2 in their nuclei. (C and D) iMOP cells also expressed (C) PAX2 and (D) ISL1. Scale bars represent 10 μm unless otherwise noted. See also Figure S5 and Table S2.

We next determined how iMOP cells undergo self-renewal by following the growth of single cells and assessing the retention of neurosensory markers. We dissociated iMOP cells into single-cell suspensions and immobilized individual cells on Matrigel to maintain their positions throughout the growth period. Single-cell cultures were allowed to proliferate and form colonies over 7 days (Figure 4A). Immunostaining showed that 92% (956/1,036) of the cells retained SOX2 labeling (n = 20) (Figure 4B). iMOP cells also maintained expression of other lineage-restricted markers. PAX2 is a neuroectoderm marker that is expressed early in otic vesicle development (at E10.5) and specifies cell types in the cochlea (Burton et al., 2004; Li et al., 2004a). ISL1 is expressed in the proneurosensory domain at E11.5, as the cochlea starts to develop and mature to form hair cells and auditory neurons (Radde-Gallwitz et al., 2004; Li et al., 2004b). We found that most of the continually proliferating iMOP cells were labeled with antibodies against PAX2 (781/852; 91.7%) and ISL1 (652/720; 90.0%), suggesting that they retained an otic neurosensory cell fate during proliferative culture (Figures 4C and 4D). As a negative control for antibody staining, clonal adherent cells derived from primary cochlear cultures expressed C-MYC, but not the proneurosensory markers observed in iMOP cells (SOX2, PAX2, and ISL1; Figure S5). The results are consistent with symmetrical self-renewal in iMOP cells.

**Figure 5:**
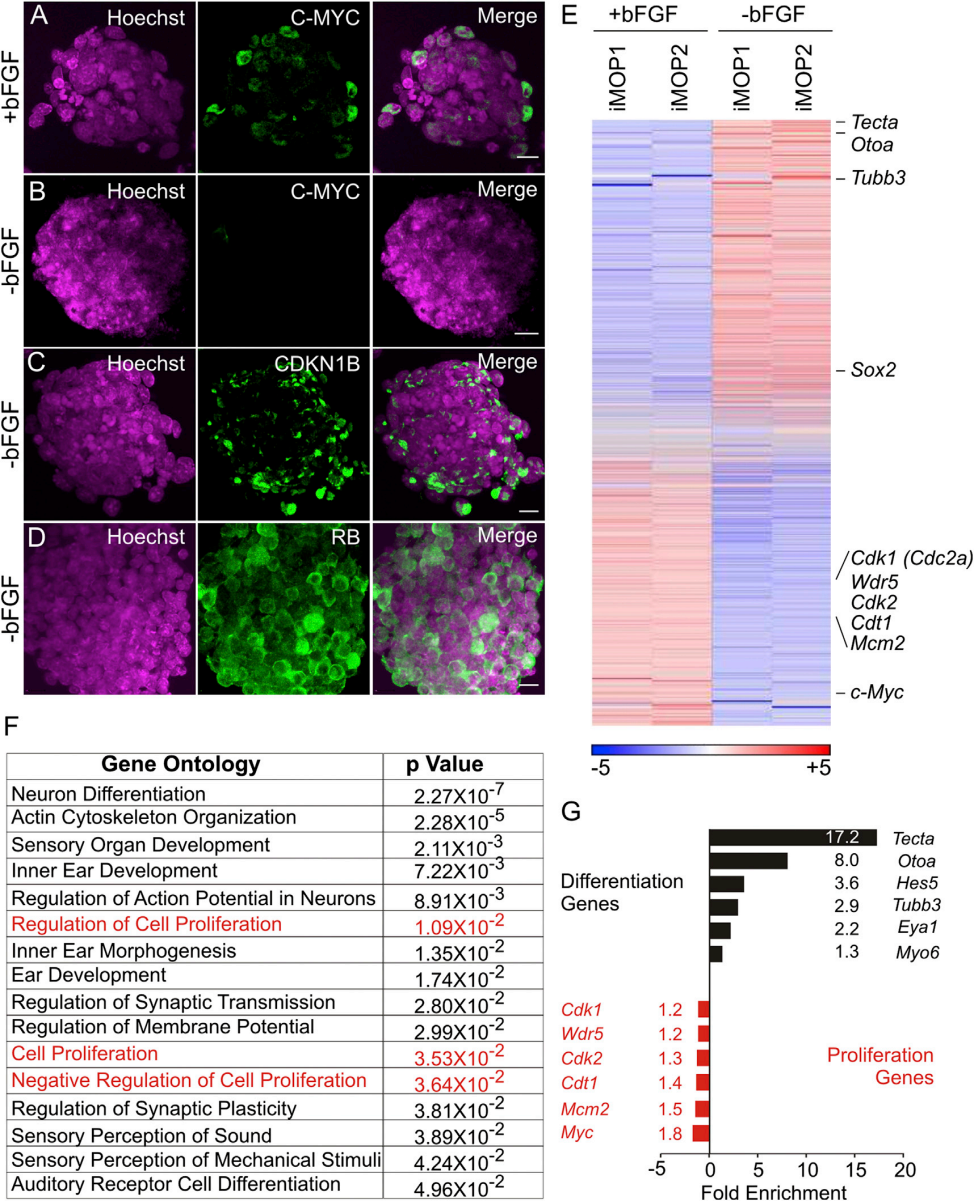
Molecular Profiling of iMOP Cells after bFGF Withdrawal (A) Nuclei of iMOP cells marked by Hoechst staining and C-MYC marked with antibody label. In the presence of bFGF, cells expressed C-MYC. (B) Removal of bFGF downregulated C-MYC expression. (C and D) Removal of bFGF also caused expression of the cell-cycle inhibitory proteins (C) CDKN1B(p27^KIP^) and (D) RB. Scale bars represent 10 μm. (E) Heatmap of all detectable genes in iMOP cells in the presence or absence of bFGF. Individual genes are listed on the right. (F) Gene Ontology analysis of differentially expressed genes (p < 0.05) revealed changes in proliferation (highlighted in red) and neuronal and otic differentiation. (G) Changes in expression levels of individual genes taken from the Gene Ontology analysis. See also Figure S6 and Table S2.

In the developing embryo, cell-cycle arrest is strongly linked to differentiation, so we asked whether treatment that leads to cell-cycle arrest promotes iMOP differentiation. iMOP cells were cultured in media lacking bFGF and containing only the B27 supplement for 7 days to monitor markers known to correspond to cell-cycle arrest in supporting cells and hair cells (Figure S6A). In the developing inner ear, supporting cells and auditory neurons express CDKN1B (p27^KIP^) after cell-cycle exit (Chen and Segil, 1999; Endo et al., 2002), and hair cells express the retinoblastoma protein RB (Sage et al., 2006). In iMOP cells, in the presence of bFGF, C-MYC was expressed and was located in the Hoechst-stained nuclei in many cells (35.2%; 361/1,025; n = 5; Figure 5A). Upon removal of growth factor for 1 day, C-MYC labeling largely disappeared from otospheres, dramatically dropping to 4.1% of cells (61/1,422; n = 5; Figure 5B). After 5 days of growth factor withdrawal, CDKN1B appeared in a punctate pattern in 48.5% (245/495) of iMOP cells (Figure 5C), and RB appeared as a diffuse signal in 30.0% (146/491; n = 5) of cells (Figure 5D). In the presence of bFGF, iMOP cells showed low levels of CDKN1B and RB expression (Figures S6C and S6D). Thus, in iMOP cells, bFGF withdrawal induced cell-cycle arrest and blocked C-MYC expression concomitantly with the induction of CDKN1B and RB expression.

To identify additional genes with altered expression after growth factor withdrawal, we conducted RNA-seq on two independently derived iMOP cell lines cultured in the presence of bFGF and two independent iMOP cell lines cultured in the absence of bFGF for 7 days. Normalized reads from significantly altered target genes (p < 0.05) were ranked and plotted on a heatmap (Figure 5E; genes of interest are noted). After bFGF withdrawal, *c-Myc* levels decreased by 1.8-fold (p < 2 × 10^−17^), consistent with decreased proliferation after growth factor withdrawal. Eight of the downregulated genes are SOX2 targets as identified by our ChIP-seq analysis. These genes (*Cdk1*, *Cdk2*, *Cdk4*, *Wdr5*, *Cdc7*, *Mcm2*, *Cdt1*, and *Skp2*) are involved in regulating cell-cycle progression and DNA replication. These data suggest that SOX2 and C-MYC together regulate transcription of genes that iMOP cell proliferation.

Along with the reduced expression of genes that modulate the cell cycle, we observed an increase in genes involved in otic differentiation. For instance, *Eya1*, which promotes early induction of otic cell fate, increased 2.2-fold (p < 0.02) (Xu et al., 1999). *Tecta* (encoding the extracellular matrix protein alpha tectorin) increased 17.2-fold (p < 10^−63^) and *Otoa* (encoding otoancorin A) increased 8.0-fold (p < 2x10^−7^). Expression of both *Tecta* and *Otoa* is restricted to the inner ear (Rau et al., 1999; Zwaenepoel et al., 2002). *Tubb3*, encoding the neuronal β-tubulin, also showed a 2.9-fold increase (p < 10^−41^). *Hes5*, a transcription factor that is activated downstream of Notch, is expressed in supporting cells, and inhibits differentiation of hair cells (Zine et al., 2001), also increased 3.6-fold (p < 10^−61^). *Myo6*, a marker for early differentiation of hair cells (Hasson et al., 1997), increased 1.3-fold (p < 10^−4^). Together, these data suggest that after growth factor withdrawal, iMOP cells begin exiting the cell cycle and start expressing genes characteristic of differentiating otic cell types, including hair cells, supporting cells, and neurons.

**Figure 6:**
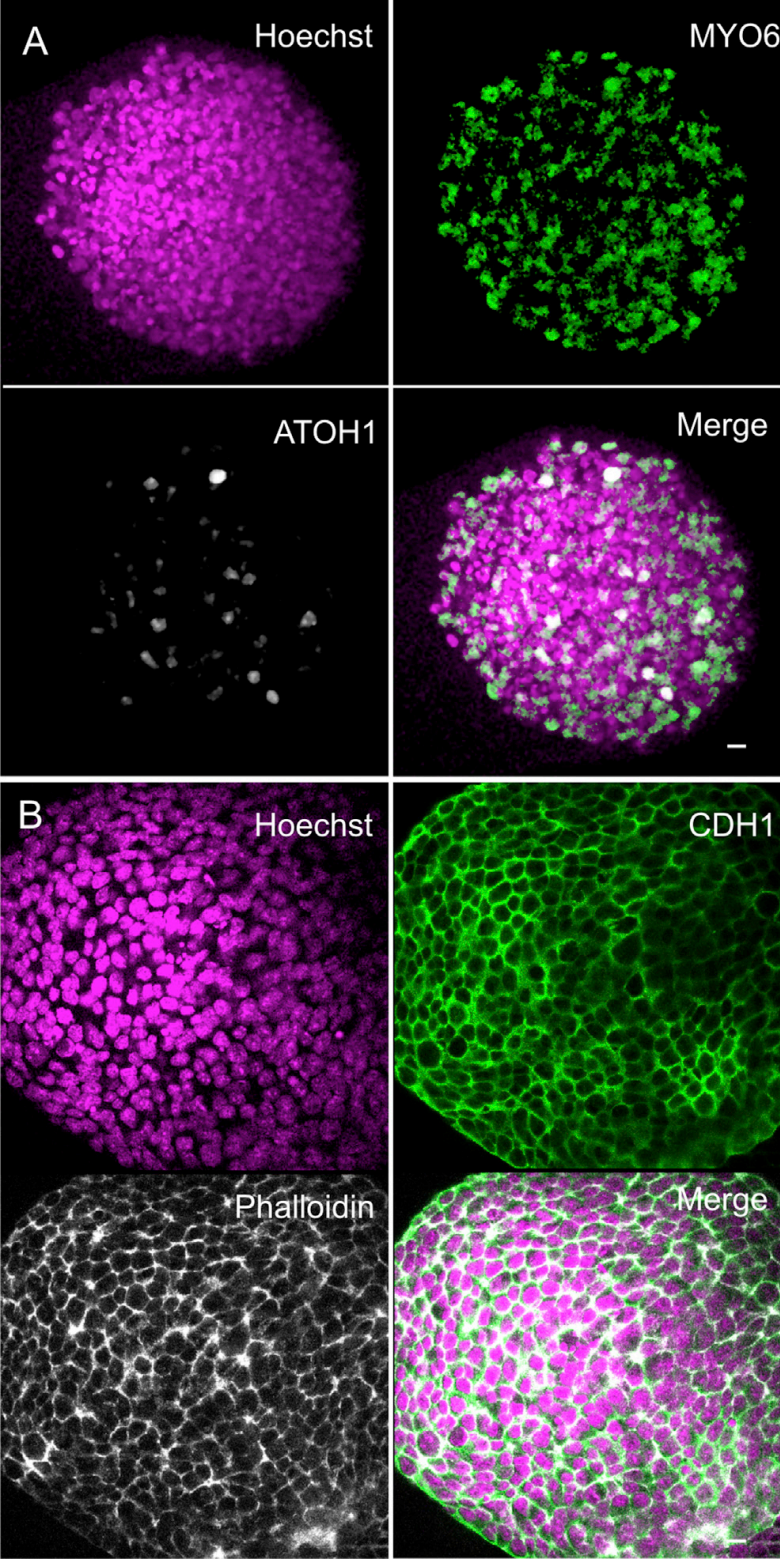
Differentiation of iMOP Cells in Suspension after bFGF Withdrawal (A) iMOP cells in an otosphere 10 days after bFGF withdrawal. Nuclei were marked by Hoechst staining and the indicated proteins were marked with antibody label. Images are merged in the last panel. Many cells showed expression of MYO6 and ATOH1. (B) Cells of another otosphere 3 days after bFGF withdrawal, showing circumferential actin bands labeled with phalloidin and adherent junctions labeled with antibodies to CDH1 (E-cadherin). Images are merged in the last panel. Scale bars represent 10 μm. See also Table S2.

When neurosensory precursors stop dividing in vivo, they start to differentiate into epithelia by forming circumferential actin bands and junctions containing CDH1 (E-cadherin) (Whitlon et al., 1999). After 10 days in culture without bFGF, iMOP cells were immunostained for MYO6, a hair cell marker, and ATOH1, a key transcription factor for hair cell differentiation (Chen et al., 2002). Of the differentiated iMOP cells, 26.3% (228/865) showed MYO6 and 11.3% (78/685) showed ATOH1 labeling (Figure 6A). These cells differentiated toward an epithelial phenotype, forming circumferential actin bands and CDH1-containing junctions as early as 3 days after bFGF withdrawal (Figure 6B).

**Figure 7:**
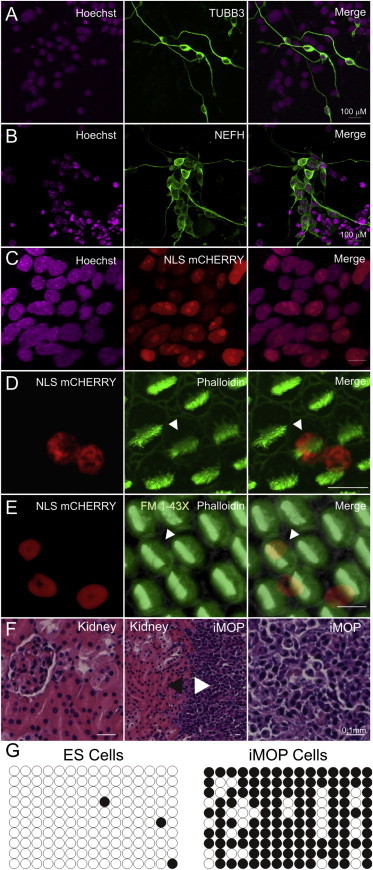
Differentiation of iMOP Cells in Different Cellular Contexts and Culture Conditions (A) iMOP cells spontaneously differentiated into neurons and are marked by TUBB3 (neuronal β-III tubulin) using a Tuj1 antibody. (B) iMOP-derived neurons are also marked by NEFH (neurofilament). (C) Nuclei of progenitor cells, marked here by Hoechst staining, were genetically labeled to express nuclear mCherry fluorescent protein. Merged fluorescence shows robust colocalization. (D) mCherry-labeled iMOP cells in chick otocysts form hair cells, as shown by phalloidin labeling of hair bundles (arrowhead), as well as supporting cells. (E) A mouse iMOP-derived hair cell (marked by arrowhead) accumulated FM1-43X to a similar extent as endogenous chick hair cells. (F) Hematoxylin and eosin staining of kidney. iMOP cells were injected into the kidney capsule and formed an encapsulated mass (white arrowhead) next to the kidney (black arrowhead). Undifferentiated iMOP cells make up the encapsulated mass. Scale bars represent 10 μm unless noted otherwise. (G) Methylation status of the *Oct4* promoter region in ESCs and iMOP cells. Each CpG dinucleotide pair along the *Oct4* regulatory region is denoted by a circle. Filled and unfilled circles correspond to methylated and unmethylated basepairs, respectively. Ten sequences were analyzed for each cell line. See also Figure S6 and Table S2.

To promote neuronal differentiation, we removed bFGF and cultured iMOP cells on an adherent laminin-coated surface (Figure S6B). After 7 days, 22.8% (54/236) of the cells developed processes that labeled with antibodies against TUBB3 (neuronal β-III tubulin) (Figure 7A). The vast majority of neuronal TUBB3-positive cells were pseudounipolar or bipolar, similar to auditory neurons. In addition, the differentiated iMOP cells were also labeled with antibodies against NEFH (neurofilament) (Figure 7B).

The cellular environment is important for regulating the iMOP cell phenotype. In vitro, hair cell differentiation after cell-cycle arrest requires additional, as yet unidentified cues provided by neighboring otic cells (Doetzlhofer et al., 2004; Oshima et al., 2010). To determine whether iMOP cells can become hair cells when provided with cues from the inner ear, we engrafted iMOP cells into developing chicken otocysts. To distinguish the mouse iMOPs from the host chicken cells, we first engineered iMOP cells using the Tol2 transposon system (Kwan et al., 2007) to express nuclear-localized mCherry fluorescent protein and the neomycin resistance gene, and selected transfected cells by culturing with neomycin. Neomycin-resistant cells showed nuclear mCherry expression (Figure 7C). Genetically modified mCherry-labeled cells were then injected into chick otocysts at E2–3 and embryos were allowed to develop until E17. At this stage of chick development, the basilar papilla (the ortholog of the cochlea in birds) contains hair cells with mature hair bundles and functional mechanotransduction (Si et al., 2003).

We found both hair cells and supporting cells derived from iMOP cells in the chicken basilar papilla. iMOP-derived cells were identified by mCherry expression and hair cells were distinguished from supporting cells by phalloidin labeling of the stereocilia (Figure 7D). In addition, hair-cell nuclei were not as deep in the epithelium as supporting cell nuclei. Among 1,192 selected cells in chicken basilar papilla, we observed 628 hair cells, 350 supporting cells, and 207 auditory neurons. Of these, 53 cells (∼5.3%) were labeled by nuclear mCherry and thus were derived from iMOPs. Of the mCherry-labeled cells, 11 were hair cells (∼21%), 30 were supporting cells (∼57%), and 12 were auditory neurons (∼23%).

To test whether the iMOP-derived hair cells were functional, we incubated freshly dissected chicken basilar papilla for 2 min in 5 μM FM1-43FX, a fixable analog of a fluorescent styryl dye that enters through functional hair-cell mechanotransduction channels (Meyers et al., 2003). The chicken basilar papilla were fixed and labeled with phalloidin to highlight the hair cells. As expected, 95% of the 691 hair cells we observed in E17 cochlea took up FM1-43FX. Twelve of them were labeled with nuclear mCherry, and 11 of the 12 accumulated the dye (Figure 7E). Thus, mouse iMOP cells, given the appropriate cues, can become bona fide hair cells. Intriguingly, the mouse hair cells in the chicken cochlea had bundles with the morphology of chicken hair cells, showing that at least some part of bundle morphogenesis is controlled by exogenous factors.

Since pluripotent cells have been described in the inner ear (Li et al., 2003), we asked whether iMOP cells, like pluripotent stem cells, can form other cell types. To address this question, we employed a teratoma-formation assay. ESC- or iPSC-derived teratomas contain cells from the three primordial germ layers (ectoderm, mesoderm, and endoderm). We transplanted iMOP cells in the kidney capsule of SCID mice and allowed them to divide and differentiate. Masses obtained from the kidney capsules were fixed in formaldehyde, embedded in paraffin, and stained with hematoxylin and eosin. We found that iMOP cells were capable of forming an encapsulated mass next to the kidney. However, histological analysis of iMOP-derived tumors did not show any differentiation, in contrast to iMOP cells grafted into the developing inner ear, and instead remained as a single homogeneous cell type (Figure 7F).

Next, we determined the methylation status of the *Oct4* promoter, which is a correlate of pluripotency of iMOP cells. In pluripotent ESCs, the promoter region of *Oct4* is unmethylated, whereas more lineage-committed cell types show varying degrees of methylation (Meissner et al., 2008). The methylation status of the *Oct4* promoter in particular is indicative of whether reprogramming of somatic cells into the pluripotent state has occurred (Fouse et al., 2008). We performed bisulfite sequencing and methylation analysis on the regulatory region of *Oct4*. The methylation pattern of CpG dinucleotides in the upstream regulatory region of the *Oct4* gene in ESCs and iMOP cells is displayed in Figure 7G. In the ten sequences used for analysis, the *Oct4* regulatory region in ESCs was vastly unmethylated, with only 2% (3/150) methylated CpG dinucleotides. In contrast, the regulatory region in iMOP cells was heavily methylated, with 80.7% (121/150) methylated CpG dinucleotides. The methylated promoter observed in iMOP cells suggests transcriptional silencing of *Oct4*. Since engraftment of iMOP cells into an inappropriate cellular environment did not result in differentiation, and the *Oct4* regulatory region is methylated, the iMOP cells apparently are not pluripotent but are lineage restricted. Only when provided with the appropriate cues from the inner ear are these progenitors able to differentiate into hair cells, supporting cells, and neurons.

## Discussion

### Induction of Self-Renewal by Transient Expression of C-MYC

The formation of otospheres has been attributed to the presence of self-renewing tissue stem cells from the inner ear. Otosphere-forming cells have a limited capacity to divide, but can become neurons and hair cell precursors (Oshima et al., 2007). We derived solid otospheres expressing SOX2, a marker for neurosensory precursor cells, from embryonic cochleas. By transiently expressing C-MYC, we activated endogenous C-MYC expression through a positive-feedback loop and amplified SOX2 targets to promote long-term self-renewal. The sustained expression of endogenous C-MYC and SOX2 accounts for the self-renewal properties of iMOP cells. Regulating the expression of genes involved in cell-cycle progression and initiation of DNA replication is a key feature of how C-MYC and SOX2 promote self-renewal and drive proliferation in iMOP cells. We propose that, unlike reprogramming of iPSCs, induction with a single factor, C-MYC, amplifies the SOX2 target genes that are responsible for self-renewal in otic cell types, but does not perturb the expression of lineage-restricted genes or the potential to differentiate.

### SOX2 and C-MYC Mediate a Transcriptional Switch from Self-Renewal to Differentiation

During cochlear development, SOX2 has been proposed to mark a common pool of precursors that is later separated into spatially distinct neurogenic domains and prosensory regions (Appler and Goodrich, 2011). Fate mapping suggests that at least some hair cells and otic neurons share a common precursor (Raft et al., 2007; Jiang et al., 2013). Zebrafish also have a common population of neurosensory progenitors that become hair cells and neurons (Sapède et al., 2012). These otic neurosensory precursors rapidly proliferate and exit the cell cycle before they differentiate. By transcriptome analysis, we showed that iMOP cells are very similar to cells from SOX2-expressing otospheres obtained from E11.5–12.5 embryonic cochleas. Using iMOP cells as a cellular platform for otic progenitors, we modeled the in vivo events of proliferation, cell-cycle arrest, and differentiation by growth factor withdrawal. We propose that at least one of the functions of C-MYC and SOX2 during the development of neurosensory precursors is to regulate proliferation and initiate differentiation.

The involvement of both C-MYC and SOX2 in promoting proliferation is consistent with the phenotype of *Sox2* hypomorphic mice. Loss of *Sox2* expression results in the absence of hair cells, supporting cells, and auditory neurons (Kiernan et al., 2005; Puligilla et al., 2010), which can be attributed to the loss of the sensory progenitors. We propose that at E11.5–12.5 of otic development, C-MYC and SOX2 are coexpressed in neurosensory precursors, and that C-MYC amplifies the transcriptional targets of SOX2, such as the cyclin-dependent kinases, to promote proliferation. We hypothesize that the lack of SOX2 alters the transcription of cell-cycle genes, prevents cell-cycle progression, and results in the loss of sensory progenitors due to the lack of cellular proliferation.

*c-Myc*, *N-Myc*, and *L-Myc* are dynamically expressed in the developing inner ear (Domínguez-Frutos et al., 2011; Kopecky et al., 2011, 2013). Although *c-Myc* mutant animals do not display any inner ear abnormalities, *N-Myc* may compensate for many of its developmental functions (Malynn et al., 2000). Deletion of *N-Myc* from the inner ear disrupts proliferation, morphogenesis, and patterning, resulting in developmental defects in both the neurosensory and nonsensory portions of the inner ear (Domínguez-Frutos et al., 2011; Kopecky et al., 2011). A recent study using conditional ablation of *L-Myc* and *N-Myc* after the formation of hair cells implicates Myc family members in the development of hair cells, as lack of *N-Myc* accelerates cell-cycle exit and delays expression of the essential transcription factor *Atoh1* (Kopecky et al., 2013). Many of the Myc family members may serve as transcriptional amplifiers during development of the inner ear to promote target genes for cell-cycle progression and even differentiation.

In early postnatal mouse utricles, a population of cells continues to divide and is a source of nascent hair cells. Ectopic expression of C-MYC in utricular cells in newborn mice leads to a mild but significant increase in proliferative capacity (Burns et al., 2012a). This may reflect increased transcriptional amplification by C-MYC in supporting cells that are already proliferating (Burns et al., 2012b). Our mechanistic proposal fits well with previous in vivo studies of how Myc family members may affect development of the inner ear.

In addition to genes that are controlled by both C-MYC and SOX2, we identified genes that are exclusively SOX2 targets. Factors other than C-MYC might regulate transcription of these SOX2 target genes. Among these are *Cdkn1a* (*p21*^*CIP*^) and *Cdkn1b* (*p27*^*KIP*^), which encode for cell-cycle inhibitors of cyclinE-CDK2 and cyclinD-CDK4/6 complexes. CDKN1A maintains quiescence in hair cells and auditory neurons (Laine et al., 2007, 2010). CDKN1B expression correlates with cell-cycle exit during differentiation of hair cells, supporting cells, and neurons, but its expression is later confined to postnatal supporting cells and spiral ganglia neurons of the cochlea (Chen and Segil, 1999; Endo et al., 2002).

Thus, in the absence of C-MYC, SOX2 may be differentially regulating *Cdkn1a* and *Cdkn1b* to exit the cell cycle and maintain quiescence. Tamoxifen-induced deletion of *Sox2* in early postnatal cochlea shows increased proliferation in inner pillar cells, but not in Deiter cells (Liu et al., 2012), suggesting that additional mechanisms may be present in cochlear cell types to maintain quiescence. We propose that *Sox2* in conjunction with the *Myc* family of genes promotes proliferation in neurosensory precursors of the developing inner ear. After cell-cycle exit and terminal differentiation, *Myc* levels are downregulated (Domínguez-Frutos et al., 2011; Kopecky et al., 2011), and *Sox2* alone may be involved in maintaining quiescence of some postmitotic cells in the cochlea.

### Conclusions

Our results indicate that a fate-restricted cell line can be generated by transient expression of C-MYC. These iMOP cells are otic-fate restricted, self-renewing, and capable of differentiating into functional hair cells and neurons. In this study, we used iMOP cells as a cellular platform to understand the role of C-MYC and SOX2 in the development of otic neurosensory precursors. Such experiments can be extended to identify additional factors required for differentiation, as well as to model genetic disorders that affect hair cells or their associated neurons. Finally, iMOP cells may join pluripotent stem cells in the repertoire of potential tools for cellular replacement therapies in the inner ear.

## Experimental Procedures

For details regarding the materials and methods used in this work, see the Supplemental Experimental Procedures. All animal work conducted was approved by the IACUC at Harvard Medical School.

## Author Contributions

K.Y.K. designed the study, performed the experiments and bioinformatics analysis, and wrote the manuscript. J.S. performed the bioinformatics analysis for RNA-seq and ChIP-seq. D.P.C. guided the study and edited the manuscript.

## Accession Numbers

The GEO accession number for the raw RNA-seq and ChIP-seq data reported in this paper is GSE62514. Analyzed ChIP-seq plots can be viewed at http://shield.hms.harvard.edu.

## Supplemental Information

Document S1. Supplemental Experimental Procedures, Figures S1–S6, and Tables S2 and S3

Table S1. Genes Bound at Their Promoters by C-MYC, SOX2, or C-MYC and SOX2 in iMOP Cells, Related to Figure 2

Document S2. Article plus Supplemental Information

## References

[bib1] Ang Y.S., Tsai S.Y., Lee D.F., Monk J., Su J., Ratnakumar K., Ding J., Ge Y., Darr H., Chang B. (2011). Wdr5 mediates self-renewal and reprogramming via the embryonic stem cell core transcriptional network. Cell.

[bib2] Appler J.M., Goodrich L.V. (2011). Connecting the ear to the brain: molecular mechanisms of auditory circuit assembly. Prog. Neurobiol..

[bib3] Burns J.C., Yoo J.J., Atala A., Jackson J.D. (2012). MYC gene delivery to adult mouse utricles stimulates proliferation of postmitotic supporting cells in vitro. PLoS ONE.

[bib4] Burns J.C., On D., Baker W., Collado M.S., Corwin J.T. (2012). Over half the hair cells in the mouse utricle first appear after birth, with significant numbers originating from early postnatal mitotic production in peripheral and striolar growth zones. J. Assoc. Res. Otolaryngol..

[bib5] Burton Q., Cole L.K., Mulheisen M., Chang W., Wu D.K. (2004). The role of Pax2 in mouse inner ear development. Dev. Biol..

[bib6] Cartwright P., McLean C., Sheppard A., Rivett D., Jones K., Dalton S. (2005). LIF/STAT3 controls ES cell self-renewal and pluripotency by a Myc-dependent mechanism. Development.

[bib7] Chen P., Segil N. (1999). p27(Kip1) links cell proliferation to morphogenesis in the developing organ of Corti. Development.

[bib8] Chen P., Johnson J.E., Zoghbi H.Y., Segil N. (2002). The role of Math1 in inner ear development: Uncoupling the establishment of the sensory primordium from hair cell fate determination. Development.

[bib9] Diensthuber M., Oshima K., Heller S. (2009). Stem/progenitor cells derived from the cochlear sensory epithelium give rise to spheres with distinct morphologies and features. J. Assoc. Res. Otolaryngol..

[bib10] Doetzlhofer A., White P.M., Johnson J.E., Segil N., Groves A.K. (2004). In vitro growth and differentiation of mammalian sensory hair cell progenitors: a requirement for EGF and periotic mesenchyme. Dev. Biol..

[bib11] Domínguez-Frutos E., López-Hernández I., Vendrell V., Neves J., Gallozzi M., Gutsche K., Quintana L., Sharpe J., Knoepfler P.S., Eisenman R.N. (2011). N-myc controls proliferation, morphogenesis, and patterning of the inner ear. J. Neurosci..

[bib12] Endo T., Nakagawa T., Lee J.E., Dong Y., Kim T.S., Iguchi F., Taniguchi Z., Naito Y., Ito J. (2002). Alteration in expression of p27 in auditory epithelia and neurons of mice during degeneration. Neurosci. Lett..

[bib13] Fantes J., Ragge N.K., Lynch S.A., McGill N.I., Collin J.R., Howard-Peebles P.N., Hayward C., Vivian A.J., Williamson K., van Heyningen V., FitzPatrick D.R. (2003). Mutations in SOX2 cause anophthalmia. Nat. Genet..

[bib14] Fouse S.D., Shen Y., Pellegrini M., Cole S., Meissner A., Van Neste L., Jaenisch R., Fan G. (2008). Promoter CpG methylation contributes to ES cell gene regulation in parallel with Oct4/Nanog, PcG complex, and histone H3 K4/K27 trimethylation. Cell Stem Cell.

[bib15] Groves A.K., Bronner-Fraser M. (2000). Competence, specification and commitment in otic placode induction. Development.

[bib16] Hagstrom S.A., Pauer G.J., Reid J., Simpson E., Crowe S., Maumenee I.H., Traboulsi E.I. (2005). SOX2 mutation causes anophthalmia, hearing loss, and brain anomalies. Am. J. Med. Genet. A..

[bib17] Hasson T., Gillespie P.G., Garcia J.A., MacDonald R.B., Zhao Y., Yee A.G., Mooseker M.S., Corey D.P. (1997). Unconventional myosins in inner-ear sensory epithelia. J. Cell Biol..

[bib18] Hotta A., Ellis J. (2008). Retroviral vector silencing during iPS cell induction: an epigenetic beacon that signals distinct pluripotent states. J. Cell. Biochem..

[bib19] Hu Z., Corwin J.T. (2007). Inner ear hair cells produced in vitro by a mesenchymal-to-epithelial transition. Proc. Natl. Acad. Sci. USA.

[bib20] Huang M., Sage C., Li H., Xiang M., Heller S., Chen Z.Y. (2008). Diverse expression patterns of LIM-homeodomain transcription factors (LIM-HDs) in mammalian inner ear development. Dev. Dyn..

[bib21] Ivanova N., Dobrin R., Lu R., Kotenko I., Levorse J., DeCoste C., Schafer X., Lun Y., Lemischka I.R. (2006). Dissecting self-renewal in stem cells with RNA interference. Nature.

[bib22] Jiang H., Wang L., Beier K.T., Cepko C.L., Fekete D.M., Brigande J.V. (2013). Lineage analysis of the late otocyst stage mouse inner ear by transuterine microinjection of a retroviral vector encoding alkaline phosphatase and an oligonucleotide library. PLoS ONE.

[bib23] Kiernan A.E., Pelling A.L., Leung K.K., Tang A.S., Bell D.M., Tease C., Lovell-Badge R., Steel K.P., Cheah K.S. (2005). Sox2 is required for sensory organ development in the mammalian inner ear. Nature.

[bib24] Koehler K.R., Mikosz A.M., Molosh A.I., Patel D., Hashino E. (2013). Generation of inner ear sensory epithelia from pluripotent stem cells in 3D culture. Nature.

[bib25] Kopecky B., Santi P., Johnson S., Schmitz H., Fritzsch B. (2011). Conditional deletion of N-Myc disrupts neurosensory and non-sensory development of the ear. Dev. Dyn..

[bib26] Kopecky B.J., Jahan I., Fritzsch B. (2013). Correct timing of proliferation and differentiation is necessary for normal inner ear development and auditory hair cell viability. Dev. Dyn..

[bib27] Kujawa S.G., Liberman M.C. (2009). Adding insult to injury: cochlear nerve degeneration after “temporary” noise-induced hearing loss. J. Neurosci..

[bib28] Kwan K.M., Fujimoto E., Grabher C., Mangum B.D., Hardy M.E., Campbell D.S., Parant J.M., Yost H.J., Kanki J.P., Chien C.B. (2007). The Tol2kit: a multisite gateway-based construction kit for Tol2 transposon transgenesis constructs. Dev. Dyn..

[bib29] Laine H., Doetzlhofer A., Mantela J., Ylikoski J., Laiho M., Roussel M.F., Segil N., Pirvola U. (2007). p19(Ink4d) and p21(Cip1) collaborate to maintain the postmitotic state of auditory hair cells, their codeletion leading to DNA damage and p53-mediated apoptosis. J. Neurosci..

[bib30] Laine H., Sulg M., Kirjavainen A., Pirvola U. (2010). Cell cycle regulation in the inner ear sensory epithelia: role of cyclin D1 and cyclin-dependent kinase inhibitors. Dev. Biol..

[bib31] Lee Y.S., Liu F., Segil N. (2006). A morphogenetic wave of p27Kip1 transcription directs cell cycle exit during organ of Corti development. Development.

[bib32] Li H., Liu H., Heller S. (2003). Pluripotent stem cells from the adult mouse inner ear. Nat. Med..

[bib33] Li H., Liu H., Corrales C.E., Mutai H., Heller S. (2004). Correlation of Pax-2 expression with cell proliferation in the developing chicken inner ear. J. Neurobiol..

[bib34] Li H., Liu H., Sage C., Huang M., Chen Z.Y., Heller S. (2004). Islet-1 expression in the developing chicken inner ear. J. Comp. Neurol..

[bib35] Lin C.Y., Lovén J., Rahl P.B., Paranal R.M., Burge C.B., Bradner J.E., Lee T.I., Young R.A. (2012). Transcriptional amplification in tumor cells with elevated c-Myc. Cell.

[bib36] Liu Z., Walters B.J., Owen T., Brimble M.A., Steigelman K.A., Zhang L., Mellado Lagarde M.M., Valentine M.B., Yu Y., Cox B.C., Zuo J. (2012). Regulation of p27Kip1 by Sox2 maintains quiescence of inner pillar cells in the murine auditory sensory epithelium. J. Neurosci..

[bib37] Malynn B.A., de Alboran I.M., O’Hagan R.C., Bronson R., Davidson L., DePinho R.A., Alt F.W. (2000). N-myc can functionally replace c-myc in murine development, cellular growth, and differentiation. Genes Dev..

[bib38] Meissner A., Mikkelsen T.S., Gu H., Wernig M., Hanna J., Sivachenko A., Zhang X., Bernstein B.E., Nusbaum C., Jaffe D.B. (2008). Genome-scale DNA methylation maps of pluripotent and differentiated cells. Nature.

[bib39] Meyers J.R., MacDonald R.B., Duggan A., Lenzi D., Standaert D.G., Corwin J.T., Corey D.P. (2003). Lighting up the senses: FM1-43 loading of sensory cells through nonselective ion channels. J. Neurosci..

[bib40] Nie Z., Hu G., Wei G., Cui K., Yamane A., Resch W., Wang R., Green D.R., Tessarollo L., Casellas R. (2012). c-Myc is a universal amplifier of expressed genes in lymphocytes and embryonic stem cells. Cell.

[bib41] Oshima K., Grimm C.M., Corrales C.E., Senn P., Martinez Monedero R., Géléoc G.S., Edge A., Holt J.R., Heller S. (2007). Differential distribution of stem cells in the auditory and vestibular organs of the inner ear. J. Assoc. Res. Otolaryngol..

[bib42] Oshima K., Shin K., Diensthuber M., Peng A.W., Ricci A.J., Heller S. (2010). Mechanosensitive hair cell-like cells from embryonic and induced pluripotent stem cells. Cell.

[bib43] Puligilla C., Dabdoub A., Brenowitz S.D., Kelley M.W. (2010). Sox2 induces neuronal formation in the developing mammalian cochlea. J. Neurosci..

[bib44] Radde-Gallwitz K., Pan L., Gan L., Lin X., Segil N., Chen P. (2004). Expression of Islet1 marks the sensory and neuronal lineages in the mammalian inner ear. J. Comp. Neurol..

[bib45] Raft S., Koundakjian E.J., Quinones H., Jayasena C.S., Goodrich L.V., Johnson J.E., Segil N., Groves A.K. (2007). Cross-regulation of Ngn1 and Math1 coordinates the production of neurons and sensory hair cells during inner ear development. Development.

[bib46] Rau A., Legan P.K., Richardson G.P. (1999). Tectorin mRNA expression is spatially and temporally restricted during mouse inner ear development. J. Comp. Neurol..

[bib47] Rebuzzini P., Neri T., Zuccotti M., Redi C.A., Garagna S. (2008). Chromosome number variation in three mouse embryonic stem cell lines during culture. Cytotechnology.

[bib48] Ruben R.J. (1967). Development of the inner ear of the mouse: a radioautographic study of terminal mitoses. Acta Otolaryngol..

[bib49] Sage C., Huang M., Vollrath M.A., Brown M.C., Hinds P.W., Corey D.P., Vetter D.E., Chen Z.Y. (2006). Essential role of retinoblastoma protein in mammalian hair cell development and hearing. Proc. Natl. Acad. Sci. USA.

[bib50] Sapède D., Dyballa S., Pujades C. (2012). Cell lineage analysis reveals three different progenitor pools for neurosensory elements in the otic vesicle. J. Neurosci..

[bib51] Si F., Brodie H., Gillespie P.G., Vazquez A.E., Yamoah E.N. (2003). Developmental assembly of transduction apparatus in chick basilar papilla. J. Neurosci..

[bib52] Stadtfeld M., Hochedlinger K. (2010). Induced pluripotency: history, mechanisms, and applications. Genes Dev..

[bib53] Suh H., Consiglio A., Ray J., Sawai T., D’Amour K.A., Gage F.H. (2007). In vivo fate analysis reveals the multipotent and self-renewal capacities of Sox2+ neural stem cells in the adult hippocampus. Cell Stem Cell.

[bib54] Takahashi K., Yamanaka S. (2006). Induction of pluripotent stem cells from mouse embryonic and adult fibroblast cultures by defined factors. Cell.

[bib55] Wernig M., Meissner A., Cassady J.P., Jaenisch R. (2008). c-Myc is dispensable for direct reprogramming of mouse fibroblasts. Cell Stem Cell.

[bib56] Whitlon D.S., Zhang X., Pecelunas K., Greiner M.A. (1999). A temporospatial map of adhesive molecules in the organ of Corti of the mouse cochlea. J. Neurocytol..

[bib57] Xu P.X., Adams J., Peters H., Brown M.C., Heaney S., Maas R. (1999). Eya1-deficient mice lack ears and kidneys and show abnormal apoptosis of organ primordia. Nat. Genet..

[bib58] Zheng J.L., Helbig C., Gao W.Q. (1997). Induction of cell proliferation by fibroblast and insulin-like growth factors in pure rat inner ear epithelial cell cultures. J. Neurosci..

[bib59] Zine A., Aubert A., Qiu J., Therianos S., Guillemot F., Kageyama R., de Ribaupierre F. (2001). Hes1 and Hes5 activities are required for the normal development of the hair cells in the mammalian inner ear. J. Neurosci..

[bib60] Zwaenepoel I., Mustapha M., Leibovici M., Verpy E., Goodyear R., Liu X.Z., Nouaille S., Nance W.E., Kanaan M., Avraham K.B. (2002). Otoancorin, an inner ear protein restricted to the interface between the apical surface of sensory epithelia and their overlying acellular gels, is defective in autosomal recessive deafness DFNB22. Proc. Natl. Acad. Sci. USA.

