# Supplementary material for: C-MYC Transcriptionally Amplifies SOX2 Target Genes to Regulate Self-Renewal in Multipotent Otic Progenitor Cells
Source: Stem Cell Reports. 2014 Dec 11;4(1):47–60. doi: 10.1016/j.stemcr.2014.11.001 (PMC4297878; doi:10.1016/j.stemcr.2014.11.001)
Supplement: Document S1. Supplemental Experimental Procedures, Figures S1–S6, and Tables S2 and S3 [file mmc1.pdf]

Stem Cell Reports, Volume *4*

Supplemental Information

C-MYC Transcriptionally Amplifies SOX2 Target Genes to Regulate Self-Renewal in Multipotent Otic Progenitor Cells

Kelvin Y. Kwan, Jun Shen, and David P. Corey

Supplemental Experimental Procedures

Dissection of Embryonic Mouse Cochleas

CD1 embryos were harvested at E11.5 or E12.5 and placed in cold HBSS without calcium or magnesium (Invitrogen). The head was bisected and the whole inner ear removed. Tissues surrounding the sensory epithelium were carefully removed. The tissue was placed in 200 µl of 0.05% TPCK-treated trypsin (Worthington Biochemicals) in HBSS for 10 minutes and mechanically triturated using fire-polished glass pipettes. Dissociation was stopped by addition of DMEM/F12 (Invitrogen) containing 10% defined FBS (Hyclone) and 25 µg/ml carbenecillin. Dissociated cells were centrifuged at 1500 rpm in a microfuge to form a pellet. The medium was removed and the cells were gently resuspended in the culture medium described below.

Culture of Primary Cells from Mouse Embryonic Cochlea and iMOP Cells

Primary cells from embryonic cochlea were cultured in DMEM/F12 (Invitrogen 11320-082) containing B27 (Invitrogen 17504-044), 20 ng/ml of bFGF (R&D Systems) and 25 µg/ml of carbenecillin at 37˚C containing 5% CO_2. Cells were trypsinized every week and expanded until they became confluent in a six-well dish. At this stage, some of the cultures formed spheres. To separate the otospheres from adherent cells, medium was gently superfused over the culture and medium containing otospheres collected. Cells were collected by centrifugation at 3,000 rpm and otospheres replated in a separate well. After 2-3 days of recovery, primary cells were used for subsequent experiments.

Induction of Self-Renewal in Proneurosensory Cells

pMX *c-Myc* IRES Puro vector was transfected into Plat-E cells (Cell Biolabs) using Fugene 6 (Roche). Virus was harvested and quantified using the QuickTiter Retrovirus Quantitation Kit (Cell Biolabs). For induction of self-renewal, primary otospheres were infected with the *c-Myc* retrovirus at a multiplicity of infection of 1-2 virus particles per cell. Clonal lines were derived by picking individual otospheres from adherent cells followed by trypsinization into single cell suspension.

Differentiation of iMOP cells

To initiate differentiation as hair cells or supporting cells, iMOP cells were cultured as floating otospheres, collected, and resuspended in medium without bFGF. Spent medium was replaced every second day or if acidified. To initiate neuronal differentiation, otospheres were mechanically titurated and plated onto 1 µg/ml poly-D-lysine and 1 µg/ml laminin. Cells were grown in culture medium without bFGF and fresh medium replenished every second day.

Injection of Chicken Otocysts with Genetically Labeled iMOP cells

Otospheres were cultured in suspension and placed in medium lacking growth factors for one day. Cells were then resuspended and gently triturated to obtain single cell suspensions, spun down and resuspended in 100 µl of medium. Fast Green was dissolved in the cell suspension to visualize the site of injection. Eggs were incubated at 37˚C in a humidified chamber for 48 hrs

before manipulations were performed. The otic vesicle was filled with cells using a pressurized Picospritzer system, visualized by Fast-Green accumulation. After the injection, the windows on the eggs were covered with a piece of tape to maintain humidity and the eggs continued incubation at 37˚C. At day 17, chicks were taken out of the eggs, the inner ear removed and fixed for immunostaining. For FM 1-43FX accumulation, the basilar papilla was attached to a coverslip using minutien pins and incubated for 2 min at room temperature in 5 μM FM 1-43FX in HBSS containing calcium and magnesium (Invitrogen). Basilar papillas were then rinsed in HBSS containing 1 μM SCAS to reduce background staining.

Immunostaining of Primary Otospheres and iMOP Cells

Cells were fixed with freshly prepared 4% formaldehyde in 1X Phosphate Buffered Saline (PBS) for 2 hr and rinsed 3 times in wash solution (1X PBS and 0.1% Triton X-100) to permeabilize the membrane, before incubating in blocking solution (1X PBS, 10% normal goat serum and 0.1% Triton X-100 for 30 minutes. Primary antibody was then added to the buffer. Primary antibodies and dilutions used for immunofluorescence are described in Supplemental Table 2. Cells were incubated in primary antibody solution from 2 hrs at room temperature to overnight at 4˚C. Cells were rinsed with cell solution and incubated with the appropriate secondary antibodies, Hoechst 33235 and phalloidin. Cells were rinsed again in wash solution with a final PBS rinse before mounting in Prolong Gold Anti-Fade (Invitrogen). Images were acquired with an Olympus FluoView 1000 scanning confocal microscope with a 63X PlanSApo objective with a NA of 1.42, at 1024x1024 resolution.

Isolation of mRNA, Preparation of cDNA and Real-Time PCR

Individual otospheres were collected and mRNA from otospheres containing primary proneurosensory cells were generated using the Ovation RNA-Seq System v2 kit (NuGEN). IMOP cells were pelleted and total RNA extracted from cells with Trireagent (Ambion). mRNA was purified from total RNA using the mRNA Direct Kit (Invitrogen) and cDNA synthesized using Superscript II Kit (Invitrogen). For real-time PCR, 2 μl of the cDNA was used in a 20 μl reaction with 300 nmol of each foward and reverse primer as well as 10 μl of the 2X Power SYBR master mix (Life Technologies) using a StepOnePlus real-time PCR machine with a two step thermocycling profile that includes a denaturation step at 95°C for 15 seconds followed by an anneal/extension step at 68°C for 30 seconds.

RNA-Seq and ChIP-Seq

For ChIP-Seq, iMOP cells were processed using conventional methods (Kim et al., 2010). For RNA-Seq, cDNA was obtained from iMOP cells as above. Libraries for deep sequencing were generated from ChIP-DNA or cDNA (Marioni et al., 2008). Single ended 36 bp Illumina GAII or HiSeq2000 raw data were obtained from Elim Biopharmeceuticals and aligned to NCBI Buid 37 (UCSC mm9) of the mouse genome using DNANexus. Each uniquely mapped read was extended 150 bp from 5′ to 3′, based on the strand of the alignment. The raw ChIP-Seq reads density across the genome in each 25 bp bin was calculated by tabulating the numbers of ChIP-seq reads within a 1kb window (+/- 500bp of mid-point) of the bin.

ChIP-Seq Density Plots of Non-Overlapping RefSeq Genes

The genomic coordinates of all RefSeq transcripts were obtained from the UCSC Genome Browser. The gene length of RefSeq transcript was calculated as the number of bases between the start position and the end position. Non-overlapping RefSeq transcripts longer than 380 bp were selected as the reference gene set. The bins covering the genomic region from 0.1 gene length upstream to the transcription start site to the end of the transcript were used to plot C-MYC, SOX2 or POLII occupancy.

Selection of High-Confidence C-MYC and SOX2 Target Genes

C-MYC and SOX2 occupancy was plotted using genomic bins within 10 kb (+/-5 kb) of the transcriptional start site of each non-overlapping RefSeq transcript. High confidence target genes were defined as those with max peak density within 2 kb (+/- 1 kb) of the transcriptional start sites and greater than the average density in the transcriptional start site proximal region in two replicated ChIP-seq experiments. Target genes for C-MYC, SOX2 or C-MYC and SOX2 can be found in Table S1.

Supplemental References

Kim, T. K. et al. Widespread transcription at neuronal activity-regulated enhancers. Nature 465, 182-187, doi:10.1038/nature09033 (2010).

Marioni, J. C., Mason, C. E., Mane, S. M., Stephens, M. & Gilad, Y. RNA-seq: an assessment of technical reproducibility and comparison with gene expression arrays. Genome Res 18, 1509-1517, doi:10.1101/gr.079558.108 (2008).

Figure S1. Harvesting and culture of solid primary otospheres containing SOX2-expressing cells. Related to Figure 1.

Cultured primary cells from otospheres expressed SOX2 and were initially proliferating as determined by incorporation of the nucleotide analog EdU.

(A) Cochleas from E12.5 embryos were dissected, dissociated with trypsin and cultured to on an untreated plate to form otospheres and adherent cells.

(B) Separation of otospheres (left) and adherent cells (right) from primary cultures.

(C) Cells from otospheres were dissociated and cultured for 2 hours in DMEM/F12, B27, 20ng/ml bFGF with 1 μM EdU before fixation. EdU was labeled with Alexafluor 594 (EdU Click-iT assay kit, Life Technologies). Cells were then labeled with SOX2 and Hoechst.

(D) Sections of otopheres from primary cultures (left) and a single otosphere at high magnification (right).

(E) Real-time PCR was used to determine expression levels of C-MYC relative to E12.5 cochlea for ES cells, progenitor cells (P) and progenitor cells two weeks after *c-Myc* retrovirus infection (P + 2 weeks). Independent experiments were done as replicates (n=3). Error bars are depicted as standard error of the mean (sem).

Figure S2. Pluripotent and proneurosensory markers in iMOP cells. Related to Figure 1.

As seen in brightfield images, (A) ES cells were cultured as adherent colonies. (B) Clonal iMOP cells were cultured in suspension as otospheres. Endogenous alkaline phosphatase activity was observed in (C) ES cells but not (D) iMOP cells. (E) Real-time PCR was used to determine the presence of *Sox2*, *Pax2* and *Isl1* transcripts in progenitor and iMOP cells. Expression levels were normalized to E12.5 cochlea. Independent experiments were done as replicates (n=3). Error bars are sem.

Figure S3. Growth curve of cells from primary otospheres. Related to Figure 1.

Cochleas from E12.5 embryos were harvested and dissociated with trypsin and cultured in media containing bFGF. Three individual primary otospheres were harvested from these mechanically titurated to dissociate cells every week. The number of cells obtained from individual otospheres after dissociation were counted. The cumulative cell number over 5 weeks from otospheres were graphed and fitted to a curve.

Figure S4. Distribution of karyotypes in iMOP cells. Related to Figure 1.

iMOP cells were cultured in colcemid to arrest cells in metaphase. Cells were harvested, treated with a hypotonic solution and dropped on to a glass slide to generate metaphase spreads. Of the 40 cells that displayed condensed metaphase chromosomes, over 50% showed a full complement of 19 pairs of autosomes and a pair of sex chromsomes. The distribution of iMOP cells that had a normal karyotype is similar to ES cells.

Figure S5. Expression of proneurosensory markers in a clonally derived proliferating adherent cell line generated from embryonic cochleas. Related to Figure 4.

A clonal adherent cell line was generated from embryonic E12.5 cochleas. The cell line was generated by *c-Myc* virus infection. The clonal cell line was cultured in the presence of bFGF and expressed (A) C-MYC but not (B) SOX2, (C) PAX2 or (D) ISL1.
Scale bars are 10 μM.

Figure S6. Expression of cell cyle inhibitors in iMOP cells in the presence of bFGF. Related to Figure 5 and 7.
(A) iMOP cells cultured as otospheres after bFGF withdrawal.
(B) iMOP cells cultured on poly-D-lysine and laminin grow as an adherent monolayer.
(C) iMOP cells cultured in bFGF rarely showed immunoreactivity for RB.
(D) Similarly, iMOP cells cultured in bFGF expressed little or no CDKN1B.

Table S1. Genes bound at their promoters by C-MYC, SOX2 or C-MYC and SOX2 in iMOP cells. Related to Figure 2

Table S2. Antibodies used for immunostaining and ChIP-Seq. Related to Figure 2,4-7.

Table S3. Primers used for RT-PCR and real-time PCR. Related to Figure 1.

Fig. S1

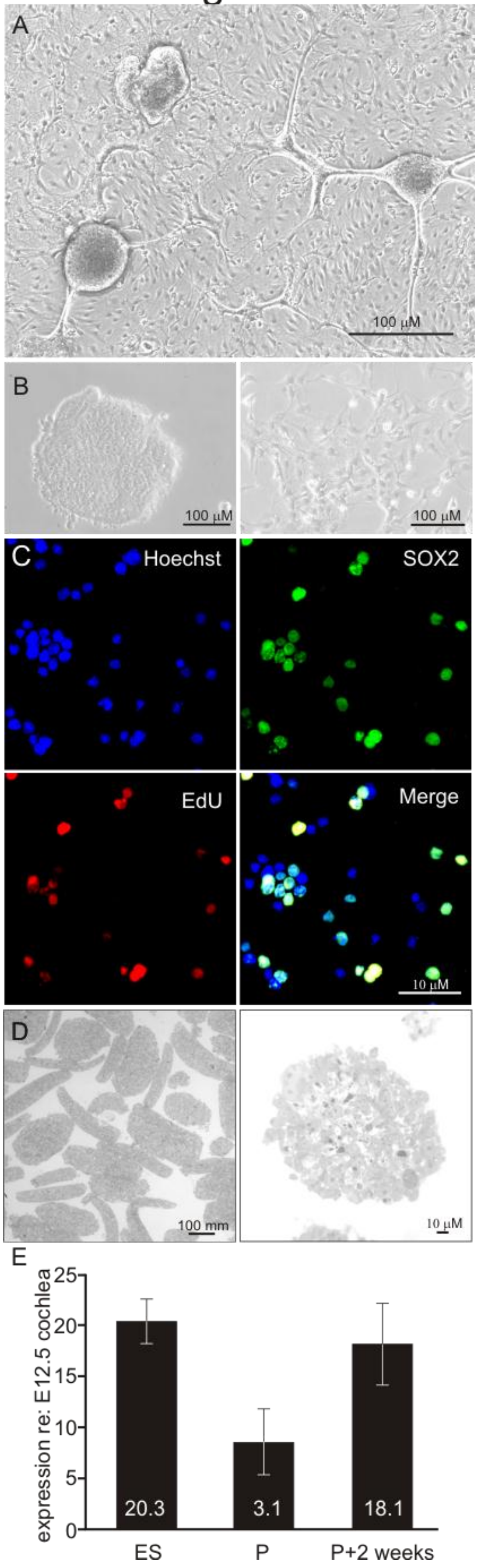

A
100 μM
B
100 μM
100 μM
C
Hoechst
SOX2
EdU
Merge
10 μM
D
100 mm
10 μM
E
expression re: E12.5 cochlea
25
20
15
10
5
0
20.3
3.1
18.1
ES
P
P+2 weeks

Fig. S2

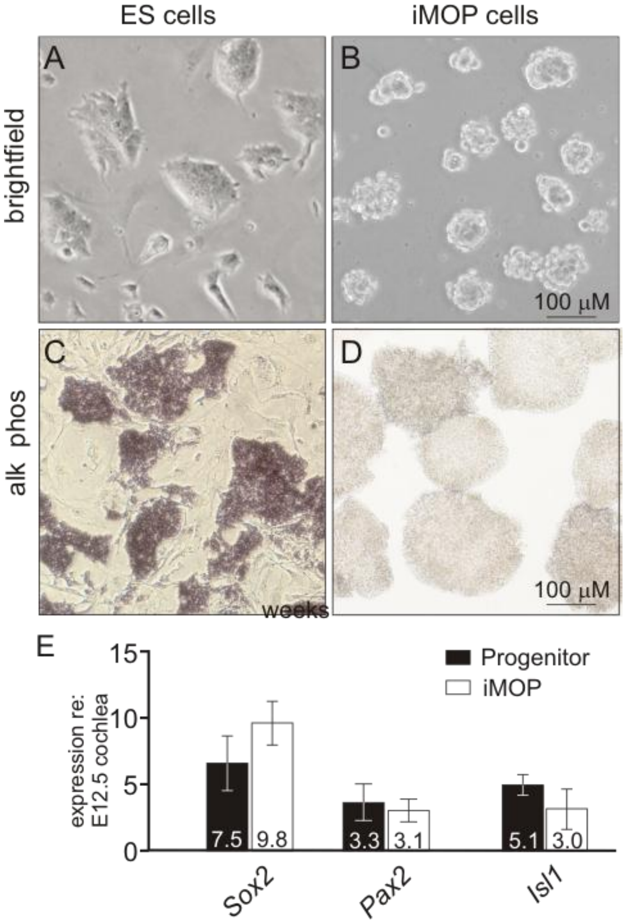
ES cells
iMOP cells
brightfield
alk phos
A
B
C
D
100 μM
100 μM
weeks
E
expression re: E12.5 cochlea
15
10
5
0
Progenitor
iMOP
7.5
9.8
3.3
3.1
5.1
3.0
Sox2
Pax2
Isl1

Fig. S3

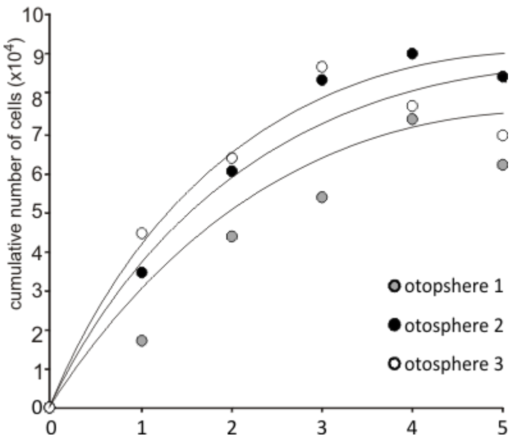

cumulative number of cells (x10^{4})
10
9
8
7
6
5
4
3
2
1
0
0
1
2
3
4
5
otopshere 1
otosphere 2
otosphere 3

Fig. S4

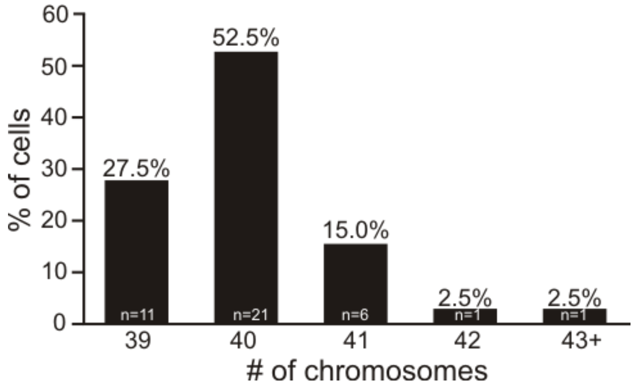

% of cells
60
50
40
30
20
10
0
52.5%
27.5%
15.0%
2.5%
2.5%
n=11
n=21
n=6
n=1
n=1
39
40
41
42
43+
# of chromosomes

Fig. S5

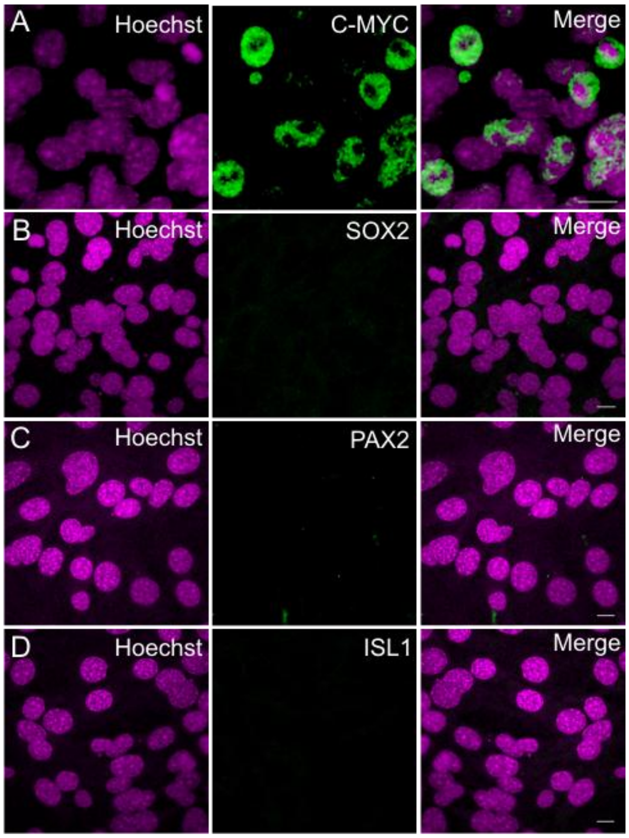

A
Hoechst
C-MYC
Merge
B
Hoechst
SOX2
Merge
C
Hoechst
PAX2
Merge
D
Hoechst
ISL1
Merge

Fig. S6

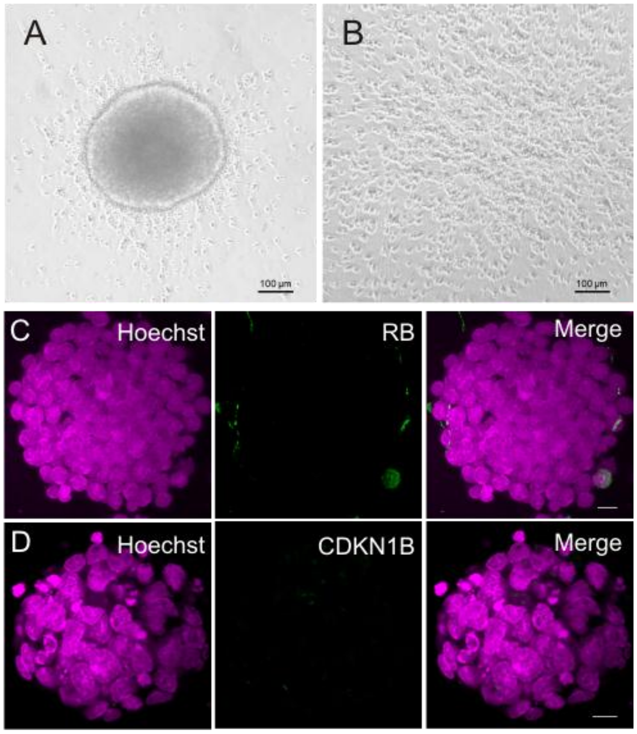

Table S2

Company	Product Number	Protein/Clone	Host	Use	Dilution/Amount Used
Proteus Biosciences	25-6791	MyoVI	rabbit	Immunostaining	1:1000
Sigma	WH0000999M1	Cdh1 clone 3F4 (E-Cadherin)	mouse	Immunostaining	1:500
Cell Signalling	9309	Rb clone 4H1	mouse	Immunostaining	1:500
Thermo Scientific	RB-9019-P0	p27Kip1	rabbit	Immunostaining	1:200
Covance	PRB-276P	Pax2	rabbit	Immunostaining	1:200
Developmental Studies Hybridoma Bank	Math1	Math1 (Atoh1) antibody	mouse	Immunostaining	1:50
Developmental Studies Hybridoma Bank	40.3A4	Isl1 antibody clone 40.3A4	mouse	Immunostaining	1:100
Chemicon/Millipore	ab1989	Neurofilament-H antibody	rabbit	Immunostaining	1:500
Chemicon/Millipore	ab5603	ChiP Antibody Sox2	rabbit	Immunostaining	1:1000
Chemicon/Millipore	17-656	ChiP Antibody hSox2	mouse	ChIP	5 µg of antibody/IP
AbCam	ab5408	RNA Polymerase II Clone 4H8	mouse	ChIP	5 µg of antibody/IP

Covance	MMS-126R	RNA Polymerase II clone 8WG16	mouse	ChIP	5 µg of antibody/IP
Santa Cruz Biotechnology	sc-764	c-Myc (N-262)	rabbit	ChIP	5 µg of antibody/IP

Table S3

**Primers for real-time PCR**	
Sox2F	ATG CAC AAC TCG GAG ATC AG
Sox2R	TGA GCG TCT TGG TTT TCC G
Pax2F	GTG GAG GTT TAC ATC TGG TCT G
Pax2R	TGA TGT GCT CTG ATG CCT G
Isl1F	CAG CAA CCC AAC GAC AAA AC
Isl1R	AGG CTG ATC TAT GTC GCT TTG
Isl2F	ATC CAC GAC CAG TTT ATC CTT C
Isl2R	CAC ACT TGA TGC CGA ACA G
Nat1F	CTA TCT TCA GAC ATC GCC AGC
Nat1R	CTA CTT GTA AAG GTG GAG CCC
GapdhsF	GTC ATG GGA GTG AAC GAG AAG
GapdhsR	CTG TGT AGG AAT GGA CTG TGG
**Primers for RT-PCR analysis**	
mc-Myc endogenous F	TGACCTAACTCGAGGAGGAGCTGGAATC
mc-Myc endogenous R	AAGTTTGAGGCAGTTAAAATTATGGCTGAAGC
mc-Myc total F	CAG AGG AGG AAC GAG CTG AAG CGC
mc-Myc total R	TTA TGC ACC AGA GTT TCG AAG CTG TTC G
mc-Myc viral F	GTGGTGGTACGGGAAATCAC
mc-Myc viral R	AGCAGCTCGAATTTCTTCCA
mOct3/4 total F	CTG AGG GCC AGG CAG GAG CAC GAG
mOct3/4 total R	CTG TAG GGA GGG CTT CGG GCA CTT
mSox2 total F	GGT TAC CTC TTC CTC CCA CTC CAG
mSox2 total R	TCA CAT GTG CGA CAG GGG CAG
mKlf4 total F	CACCATGGACCCGGGCGTGGCTGCCAGAAA
mKlf4 total R	TTAGGCTGTTCTTTTCCGGGGCCACGA
mNat1 F	ATTCTTCGTTGTCAAGCCGCCAAAGTGGAG
mNat1 R	AGTTGTTTGCTGCGGAGTTGTCATCTCGTC
